# Cross-sectional study of calves from Norwegian fattening herds with enzootic pneumonia: pathogen occurrence, clinical relevance, antimicrobial resistance, and agreement between respiratory tract sampling sites

**DOI:** 10.3389/fvets.2026.1824642

**Published:** 2026-06-24

**Authors:** Lise Marie Ånestad, Silje Enge Falkeid, Veslemøy Sunniva Oma, Randi Therese Garmo, Amelia R. Woolums, Ane Mohn Bjelland, Maria Stokstad, Thea Blystad Klem

**Affiliations:** 1 Norwegian Veterinary Institute, Ås, Norway; 2 Department of Production Animal Clinical Sciences, Norwegian University of Life Sciences, Ås, Norway; 3 Department of Research and Development, Farm Advisory Services, TINE SA, BTB-NMBU, Ås, Norway; 4 Department of Pathobiology and Population Medicine, Mississippi State University, Mississippi State, MS, United States; 5 Department of Bacteriology, Norwegian Institute of Public Health, Oslo, Norway

**Keywords:** antimicrobial susceptibility, bovine respiratory disease (BRD), bronchoalveolar lavage (BAL), diagnostics, *Mannheimia haemolytica*, nasal swab, nasopharyngeal swab, *Pasteurella multocida*

## Abstract

Bovine respiratory disease (BRD) is a leading cause of morbidity and antimicrobial use in calves. Laboratory diagnostics are limited by difficulty obtaining lower airway samples from live animals and the common presence of many BRD-associated bacteria in healthy calves, complicating culture interpretation. This study aimed to investigate the occurrence, clinical relevance, and antimicrobial resistance patterns of BRD-associated bacteria in calves from Norwegian fattening herds with enzootic pneumonia, and to evaluate agreement between upper and lower airway sampling sites. In a cross-sectional study, 88 calves from seven fattening herds were clinically scored and classified as healthy or diseased. Nasal swabs, nasopharyngeal swabs, bronchoalveolar lavage (BAL) fluid, and serum samples were collected for bacterial culture, antimicrobial susceptibility testing by disc diffusion, and serology. *Pasteurella multocida* and *Mannheimia haemolytica* were the most frequently detected bacteria across sampling sites, while *Histophilus somni* was less common. In BAL samples, *P. multocida* was the predominant bacterium identified and was significantly associated with clinical disease. Agreement between upper airway samples and BAL for *Pasteurellaceae* detection was slight to fair at the individual level. However, group-level isolation rates for *P. multocida* were similar across sampling sites in diseased calves. Most isolates were susceptible to common BRD antimicrobials, although penicillin-resistant *M. haemolytica* was detected in 18% of *M. haemolytica*-positive calves. Antibodies against *Mycoplasmopsis bovis* were not detected. In conclusion, *P. multocida* appears to be the bacterial pathogen most strongly associated with clinical disease in calves from Norwegian fattening herds with enzootic pneumonia. Upper airway sampling had limited value for individual diagnosis but may be useful for herd-level assessment when diseased calves are sampled. Antimicrobial resistance levels were generally low, although resistance was detected.

## 1 Introduction

Respiratory tract infections, often referred to as bovine respiratory disease complex (BRD), represent the most important health challenge in calves and young stock worldwide (1). Across all cattle production systems, BRD causes substantial morbidity and mortality, and long-term reductions in growth and productivity (2–4). Internationally, large amounts of antimicrobials (AM) are used for treatment and control, contributing to the emergence of multi-resistant bacteria (5–7). Effective management is further complicated by the multifactorial nature of BRD, where environmental stressors, host immunity factors, and multiple pathogens interact to cause pneumonia. Calves in fattening units are particularly vulnerable, as transport, regrouping, and commingling of animals from different origins increase their risk of developing disease (8).

Pathogenesis of BRD typically begins with immunosuppression from stress or primary viral infection, followed by secondary bacterial invasion (1). Frequently involved bacteria include *Pasteurella multocida, Mannheimia haemolytica*, and *Histophilus somni*, members of the *Pasteurellaceae* family that are generally considered commensals of the upper airways but capable of lung infection, usually under predisposing conditions (9). *Mycoplasmopsis bovis* (formerly *Mycoplasma bovis*) is another globally important pathogen. It has not been detected in Norwegian livestock (10–12), but has emerged in neighboring countries over the past two decades (13–15). Other opportunistic bacteria, such as *Trueperella pyogenes* and *Bibersteinia trehalosi*, may occasionally be involved in BRD (16). Although many viruses are often considered primary pathogens and bacteria secondary, this distinction is often uncertain, and pathogenicity may vary between bacterial species and strains (8, 17). Improved knowledge of bacterial species present in herds affected by BRD is necessary to better understand BRD pathogenesis and virulence among circulating strains, and to guide appropriate AM therapy.

Because bacteria often contribute to BRD, AMs are routinely used for treatment. In recent years, a tendency of increasing AM resistance (AMR) among BRD-associated bacteria has been reported in several countries across Europe and North America (7, 18–20). In many regions, critically important AMs, such as amphenicols and macrolides classified under European Medicines Agency (EMA) category C, are often used instead of narrow-spectrum drugs like procaine benzylpenicillin (EMA category D), despite the greater selection pressure and resistance associated with broader-spectrum agents (6). This widespread use may partly reflect the availability of single-dose therapies and long-acting formulations, and the prevalence of *M. bovis*, which is naturally resistant to penicillin. In Norway, where *M. bovis* is presumed absent, procaine benzylpenicillin is recommended as the first-choice AM (21). However, few systematic studies of BRD-associated bacteria and their AMR pattern have been done in Norway to strengthen the evidence base for these guidelines. Ideally, such studies would target herds prone to high AM use, where the likelihood of detecting AMR is greatest, such as fattening herds with long-term clinical problems. Current knowledge relies mainly on the limited number of clinical and post-mortem samples submitted to diagnostic laboratories.

Although identifying causative bacteria and their AMR pattern is essential for prudent AM use, most clinicians treat pneumonic calves empirically, without laboratory analyses. This is partly due to the diagnostic uncertainty of BRD bacteriology, given that BRD bacteria may act as commensals or opportunistic pathogens and are commonly detected in both the upper and lower airways of healthy calves (8, 22, 23). Thus, detection does not necessarily indicate infection or causation. Because impaired respiratory defenses may favor bacterial proliferation, bacterial quantification has been proposed as a way to distinguish infection from commensal colonization, with higher bacterial amounts potentially associated with clinical disease. However, studies comparing bacterial amounts between healthy and diseased calves have reported variable results (24–26), and further investigation is needed.

Selecting the most appropriate sampling site for detecting BRD pathogens is another challenge. Nasal swabs (NS), guarded nasopharyngeal swabs (NPS), and bronchoalveolar lavage (BAL) are commonly used methods, each with advantages and limitations. NS and NPS are less invasive than BAL, but their ability to represent lung pathogens is uncertain, and interpretation may be complicated by polymicrobial overgrowth. BAL, in contrast, provides a lung-derived sample with far less polymicrobial overgrowth and is therefore easier to interpret (23). Although BAL is not a true gold standard, it remains one of the best available proxies for lower airway infection under field conditions. Upper airway sampling would be preferable from an animal welfare and practical perspective; however, agreement between upper and lower airway samples varies across studies (22, 23, 27–30), leaving no clear consensus on the diagnostic value of NS and NPS for representing lung pathogens.

To address these knowledge gaps, this study aimed to improve understanding of BRD-associated pathogens, their clinical relevance, and the diagnostic value of different sampling sites in calves from Norwegian fattening herds with respiratory problems over time. Our specific objectives were to (1) investigate the occurrence and phenotypic AMR pattern of BRD-associated pathogens, including the presence of bacterial co-occurrences and viruses; (2) compare semi-quantitative culture results from NS, NPS, and BAL samples between healthy and diseased calves; and (3) assess the agreement and predictive values of NS and NPS relative to BAL for detecting BRD pathogens in healthy and diseased calves.

## 2 Materials and methods

### 2.1 Study design

#### 2.1.1 Overall design

This cross-sectional study included calves from Norwegian fattening herds with enzootic pneumonia, located in Southern Norway. Material collection included farm data, individual calf clinical examination, and collection of nasal swabs, nasopharyngeal swabs, bronchoalveolar lavage, and serum samples at the time of the visit. Herd visits took place between October 2021 and March 2022, with each herd visited once at a randomly selected time. For one analysis, data from a parallel study on dairy calves conducted during the same period were included in order to improve analytical power. Details about the study of dairy calves are presented by Ånestad et al. (31).

Laboratory analyses were performed at the Norwegian University of Life Sciences (NMBU) and the Norwegian Veterinary Institute (NVI). The study was approved by the National Animal Research Unit in the Norwegian Food Safety Authority (NFSA) under license FOTS ID 26090, and informed consent was obtained from all farms. The calf was the study unit. This study was primarily exploratory, but also powered for selected predefined, clinically relevant comparisons. Sample size was constrained by practical and logistical factors including herd availability, personnel resources, and the time-intensive, invasive nature of the sampling procedures. Post-hoc sample size considerations are described below.

#### 2.1.2 Selection of herds

Fattening herd candidates purchased calves for fattening until slaughter and had herd sizes larger than the 2020 average (>59.8 calves) for herds without dams in the Beef Cattle Performance Recording System. Selected herds also experienced long-term problems with BRD, confirmed by treatment registration data and information from farmers and veterinarians. The animals in these herds were mainly dairy bull calves bought shortly after weaning (at 2–3 months old) and slaughtered at around 9 months.

Lists of fattening farms meeting the inclusion criteria were provided by Animalia, a Norwegian livestock industry research center. Farms were selected based on consistent year-round BRD diagnoses in calves, convenient access, and location in regions with high cattle farm density. The dairy herds included in parts of this study also had persistent BRD-issues, with selection criteria described by Ånestad et al. (31).

#### 2.1.3 Selection of individuals and housing conditions

Calves older than 10 days were eligible for enrollment, both with and without respiratory signs. Exclusion criteria included AM treatment or vaccination within 14 days prior to the visit, or severe dyspnea making sedation unsafe. Due to the small herd sizes, the number of available calves was often limited. For practical purposes, the youngest calves were prioritized. As many calves as possible were sampled during the visit time (median: 12; range 4–20), usually from the same pen or neighboring pens. Calves were kept in typical Norwegian housing systems; in groups of 3–15 on either slatted floors, deep litter, or straw bedding. Pre-weaned calves were fed milk replacer or fresh milk via automatic milk feeders or milk bars, while weaned calves received silage and concentrate.

#### 2.1.4 Final study population

The final study population included seven fattening herds (farm IDs J to P), located in the south-east (*n* = 5), south-west (*n* = 1), and north-west (*n* = 1) parts of Southern Norway. From these herds, a total of 88 calves were selected (11 heifers and 77 bull calves), of which 82 were Norwegian Red, five were crossbreeds, and one was Charolais. The mean age was 135 ± 45 days (median: 138; range: 20–257), with 10 pre-weaned and 78 post-weaned calves. Four herds (J, L, M, and P) vaccinated against bovine respiratory syncytial virus (BRSV), bovine parainfluenza virus type 3 (BPIV3), and *M. haemolytica* using an inactivated injectable vaccine (Bovilis Bovipast RSP Vet., MSD Animal Health).

The AM treatment routines varied between study herds. In the years prior to sampling, BRD was usually treated as follows: Farms N, O, and P used procaine benzylpenicillin exclusively; farm K used either procaine benzylpenicillin or oxytetracyline; farm J used florfenicol; farm L used amoxicillin or trimethoprim-sulfa; and farm M used gamithromycin.

An additional 131 calves (68 heifers and 63 bull calves) from nine dairy herds (farm IDs A-I) in a parallel study were included in one analysis; the assessment of agreement between upper and lower airway sampling sites in healthy vs. diseased calves. Their inclusion ensured sufficient sample size within each health group. Details on the dairy calves are described by Ånestad et al. (31). Further information on all study herds and calves is provided in Supplementary Table S1.

### 2.2 Collection of data and biomaterial

**Figure 1:**
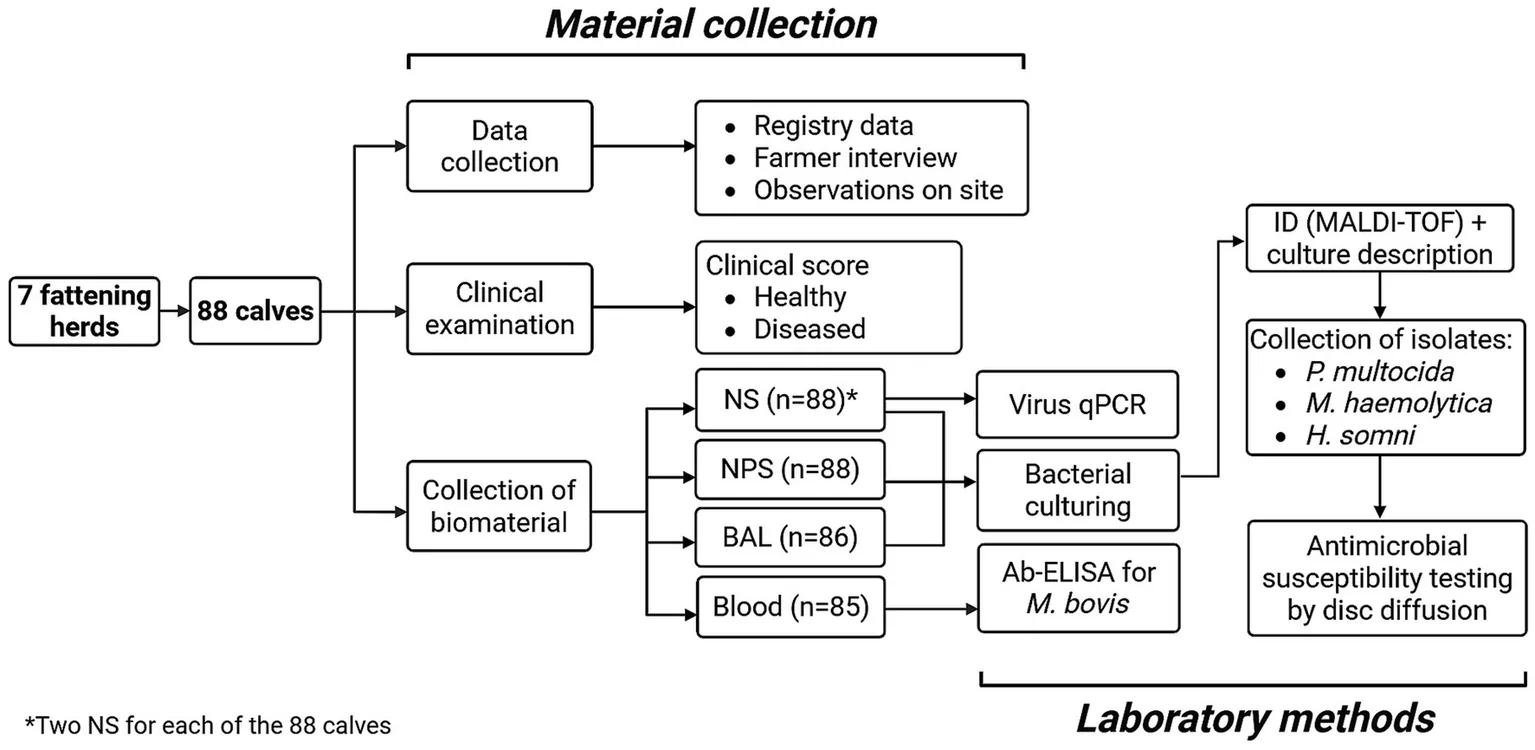
Overview of data and material collection procedures. The main study population consisted of calves from fattening herds. Collection of material was done in the same manner in all herds, regardless of production type. For NS, two swabs were collected from each calf: one for virus qPCR and one for bacterial culturing. For all other sample types, a maximum of one sample per calf was collected. NS, nasal swab; NPS, nasopharyngeal swab; BAL, bronchoalveolar lavage. Figure created using BioRender.com, modified from Ånestad et al. (31), originally published in BMC Veterinary Research under the CC BY 4.0 license.

Figure 1 provides an overview of data and biomaterial collection. A summary of procedures follows below.

#### 2.2.1 Data collection

We collected data on calf identity (birth, gender, breed) and health history from the Norwegian Dairy Herd Recording System and on-farm records, and AM treatments (drug, date, and duration) from the Norwegian Animal Health Recording System. Additional information regarding herd health, management practices, and AM treatment protocols was collected through farmer interviews and on-site observations. Questionnaire forms used are provided in Supplementary Tables S2, S3. Similar forms were used in dairy herds (31).

#### 2.2.2 Clinical examination

All calves were examined by the same experienced veterinarian using a clinical scoring system adapted from Klem et al. (32), originally developed for acute viral pneumonia and modified to include chronic BRD (31). The assessment included respiratory rate, rectal temperature, cough, nasal discharge, lung auscultation, and general appearance (Table 1). Each parameter contributed with 0–3 points, giving a total score ranging from 0 to 18. Calves scoring above five were classified as diseased. The cut-off was based on signs that would typically lead to a BRD diagnosis in field practice.

**Table 1:** Percentages (with counts) of clinical findings and corresponding scores among 88 fattening calves from seven herds and 131 dairy calves from nine herds.

Score	Production type	Respiratory rate (breaths/min)	Rectal temperature (°C)	Cough^1^	Nasal discharge	Lung auscultation^2^^,^^3^	General appearance^4^
0		≤39	≤39.5	No cough observed	Normal	Normal	Bright, alert
	Fattening	57% (50)	72% (63)	34% (30)	16% (14)	20% (18)	84% (74)
	Dairy	53% (70)	87% (114)	44% (57)	21% (28)	49% (64)	76% (100)
1		40–55	39.6–39.9	Induced cough	Serous		Mildly depressed
	Fattening	32% (28)	18% (16)	6% (5)	34% (30)	—	14% (12)
	Dairy	34% (44)	8% (11)	6% (8)	34% (45)	—	20% (26)
2		56–70	40.0–40.4	1 sporadic cough	Mucous		Moderately depressed
	Fattening	9% (8)	8% (7)	22% (19)	32% (28)	—	2% (2)
	Dairy	11% (14)	2% (3)	11% (14)	24% (32)	—	3% (4)
3		>70	>40.4	>1 sporadic cough	Mucopurulent or purulent	Abnormal sounds	Severely depressed
	Fattening	2% (2)	2% (2)	39% (34)	18% (16)	80% (70)	0% (0)
	Dairy	2% (3)	2% (3)	40% (52)	20% (26)	51% (67)	1% (1)

Clinical scores were based on a scoring system modified from Klem et al. For each clinical parameter, calves were scored from 0 to 3, resulting in a total clinical score (CS) ranging from 0 to 18. Calves with CS > 5 were classified as diseased.

1 Cough was recorded during the approximately 10-min-long individual clinical examination.

2 Auscultation was performed at seven different sites of the lung field; dorsal, middle, and ventral on the left and right side, and under the triceps muscle on the right side.

3 Abnormal sounds included crackles, wheezes, and pathologically increased or decreased respiratory sounds.

4 General appearance was categorized as follows: bright and alert, mildly depressed (standing, calmer than usual), moderately depressed (prefers lying down, stands up in response to contact), or severely depressed (lying down, minimal or no response to contact).

#### 2.2.3 Collection and storage of biomaterial

Sampling procedures were identical to those described by Ånestad et al. (31). In brief, after the clinical examination, we sedated selected calves and sampled them in sternal recumbency. We sequentially collected nasal swabs (NS), guarded nasopharyngeal swabs (NPS), non-endoscopic bronchoalveolar lavage (BAL) fluid, and blood samples. One NS was collected from each nostril for bacterial culture (Eswab™490 CE. A, Copan, Brescia, Italy) and viral qPCR (Floqswabs®502CS01, Copan, Brescia, Italy), respectively, followed by an NPS (Laryngeal swab™MW128, Medical Wire, Wilts, England) from the nasopharynx. BAL fluid was collected using a custom-made BAL catheter (23) inserted through a locally anesthetized nostril and wedged into a bronchus. Sixty milliliters of sterile saline 0.9% was instilled and aspirated, returning fluid with a foam layer. The procedure was repeated once if fluid recovery was unsuccessful. Blood was collected from the external jugular vein.

Samples for bacterial culture (NS, NPS, and BAL) were stored at 4 °C and processed within 24 h. NS samples for viral qPCR were snap-frozen on dry ice and stored at −80 °C within 24–48 h. Blood was kept at room temperature for 1–3 h, then at 4 °C until centrifugation, and serum aliquots were stored at −80 °C until analysis.

### 2.3 Laboratory analyses and handling of samples

#### 2.3.1 Bacterial culture and identification

Bacterial culture procedures were performed at NMBU as described previously (31). Briefly, NS, NPS, and 1 μL of vortexed BAL fluid were cultured on 5% bovine blood agar (prepared at NVI, Ås, Norway) in three dilutions. BAL samples (1 mL) were also enriched with brain heart infusion (BHI) broth, which were plated only if primary BAL cultures showed no growth. Plates were incubated overnight under appropriate atmospheric conditions. All plates were assessed semi-quantitatively by the same investigator. Suspected BRD-associated bacteria were sub-cultured and identified using matrix-assisted laser desorption/ionization time of flight mass spectrometry (MALDI-TOF MS) with the VITEK®MS system and V3.2 database (bioMérieux, Craponne, France). One representative colony per species per sampling site was selected. All confirmed isolates of *P. multocida*, *M. haemolytica*, and *H. somni* (collectively referred to as *Pasteurellaceae* spp. in this study), were subjected to AM susceptibility testing.

#### 2.3.2 Antimicrobial susceptibility testing

Antimicrobial susceptibility testing was performed using disc diffusion, according to the European Committee on Antimicrobial Susceptibility Testing (EUCAST) protocol, as described previously (31). The AM panel included penicillin, amoxicillin-clavulanic acid, trimethoprim + sulfamethoxazole, tetracycline, enrofloxacin, florfenicol, and streptomycin. Streptomycin, while included in the panel, was not included in the definition of commonly used BRD AMs. Inhibition zones (Supplementary Table S4) were interpreted using breakpoints from EUCAST when available; otherwise, those of the Clinical and Laboratory Standards Institute (CLSI) were applied. Isolates were classified as susceptible (S), susceptible at increased exposure (I), or resistant (R).

#### 2.3.3 Virus identification

At NMBU, viral RNA was extracted from NS using the NucliSens® miniMAG™ system (bioMérieux) following the manufacturer’s instructions. Multiplex real-time PCR (qPCR; Pneumo 4BV kit, DNA diagnostic A/S, Risskov, Denmark) was used to detect BPIV3, bovine coronavirus (BCoV), BRSV, bovine viral diarrhea virus (BVDV), and bovine herpesvirus 1 (BHV1). Samples with a cycle threshold (Ct) value < 37 were considered positive.

#### 2.3.4 Antibody detection for *Mycoplasmopsis bovis*

Serum samples were analyzed at NVI for the presence of antibodies against *M. bovis* using an indirect ELISA kit (IDScreen® *Mycoplasma bovis* Indirect, IDvet, Grabels, France), as described previously (31). Samples were run in duplicate and interpreted according to the manufacturer’s instructions.

### 2.4 Data management and statistical analyses

#### 2.4.1 Categorization and data management

**Figure 2:**
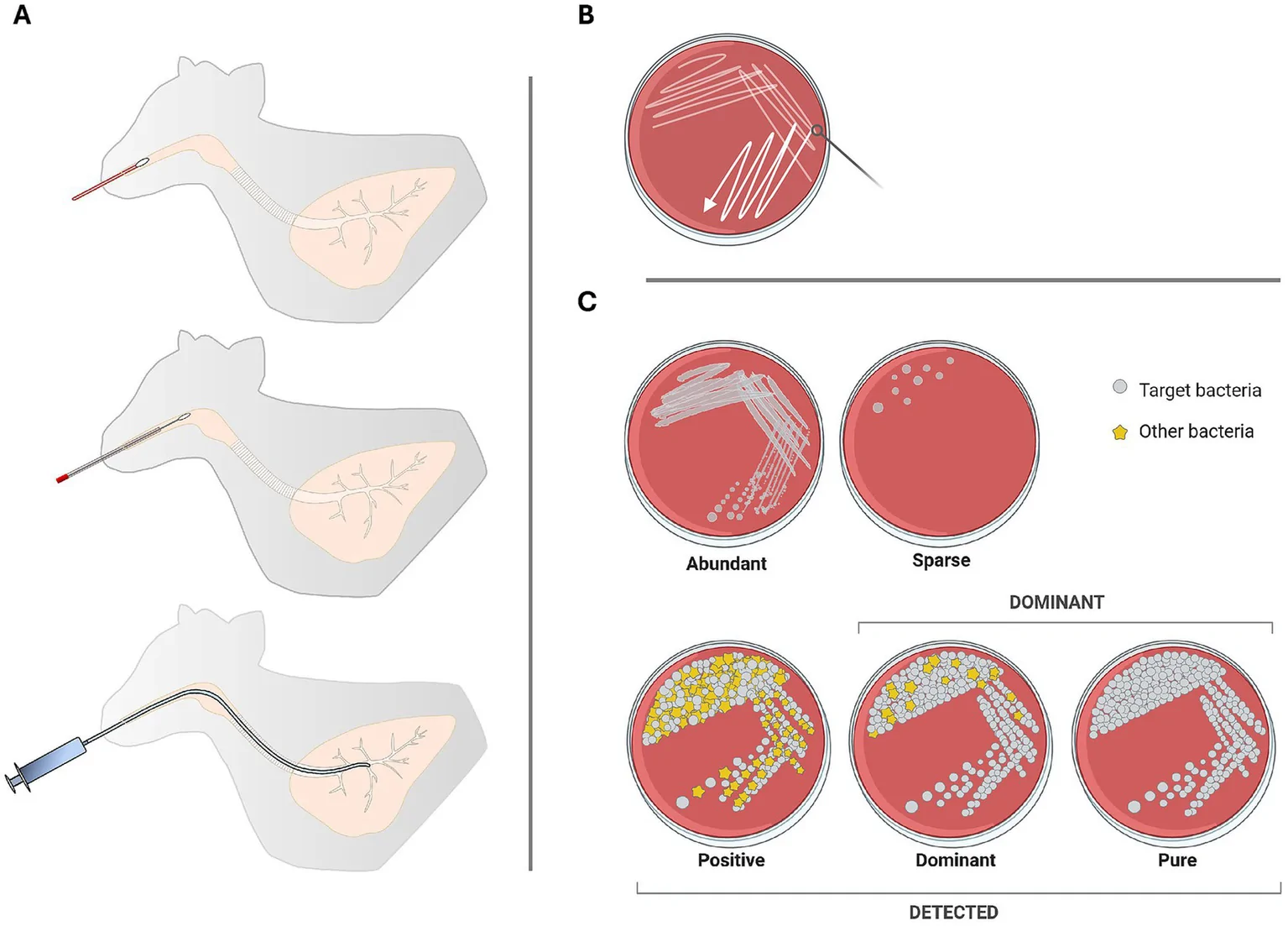
Sampling procedures **(A)** included (from top to bottom) nasal swabs, nasopharyngeal swabs, and bronchoalveolar lavage samples, followed by conventional bacterial culturing in three dilutions **(B)** and semi-quantitative recording of bacterial species and growth **(C)**. In panel C, the top two cultures illustrate how we described the abundance of growth in each culture, while the bottom three show how we described the composition of those cultures. For analyses, culture results were combined into broader categories (indicated in capital letters), defined as follows: “detected” = all cultures where target species were present (positive + dominant + pure); “dominant” = dominant and pure cultures, and cultures with ≥ 2 *Pasteurellaceae* spp. exclusively with equal growth (not shown in illustration); and “dominant and abundant” = cultures with abundant growth within the “dominant” category. Illustration: Lise Marie Ånestad using Gravit Designer **(A)** and BioRender.com **(B,C)**, the latter adapted from Ånestad et al. (31), originally published in BMC Veterinary Research under the CC BY 4.0 license.

Culture results included the following definitions: negative (no colonies); positive (≥ 1 colony); dominant (≥ 1 species outgrowing others); and pure (only 1 species) cultures (Figure 2). Co-occurrence was defined as ≥ 2 *Pasteurellaceae* spp., each with ≥ 1 colony. Growth in NS and NPS cultures was classified as sparse (presence in ≤ half of the first dilution) and abundant (> half of first dilution); in BAL, sparse was < 10 colonies and abundant ≥ 10. Contamination in BAL was defined as growth with < 10 colonies and ≥ 3 species without dominance, or dominance by non-pathogenic flora. Enriched and non-enriched BAL cultures were analyzed separately.

All outcomes were binary. Culture results were categorized at three levels: (1) detected (any presence), (2) dominant (dominant, pure, or equal growth of *Pasteurellaceae* spp. only), and (3) dominant with abundant growth. Animals were positive if ≥ 1 sampling site (NS, NPS, or BAL, including enriched samples) was positive; and herds were positive if ≥ 1 animal tested positive. Antimicrobial susceptibility categories (S, I, and R) were each analyzed separately. Clinical and laboratory data were merged only after all results were final to prevent bias.

#### 2.4.2 Statistical analyses

**Figure 3:**
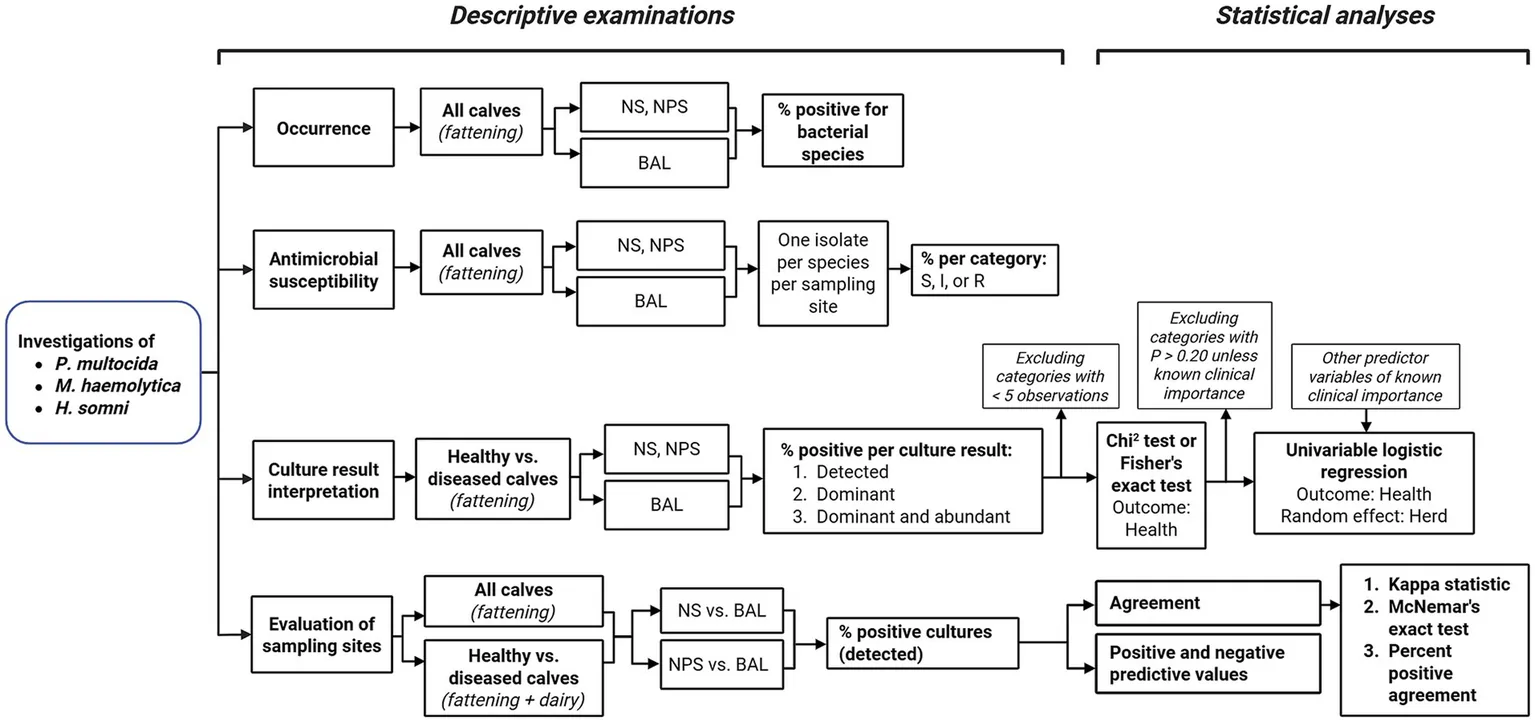
Overview of the analytical approach for *Pasteurellaceae* spp. culture results. Descriptive and statistical analyses were performed either on all calves combined or on healthy and diseased calves separately. For evaluation of sampling sites by health status, data from a study of dairy calves (Ånestad et al. (31)), which was completed at the same time as the present study, were also included to increase statistical power. Upper airway sites were represented by NS and NPS (analyzed separately), and the lower airway site by BAL. For culture result interpretation analyses, each category (e.g., dominant cultures) was evaluated for each sampling site. Smaller subcategories (e.g., pure cultures) were also analyzed if they occurred in ≥ 5 calves. Variables with < 5 total observations (among all calves) were not tested. Pearson’s chi-squared test was used when each health group had ≥ 5 observations; Fisher’s exact test was applied if at least one group had < 5 observations. NS, nasal swab; NPS, nasopharyngeal swab; BAL, bronchoalveolar lavage; S, susceptible; I, susceptible, increased exposure; R, resistant. Figure created using BioRender.com and adapted from Ånestad et al. (31), originally published in BMC Veterinary Research under the CC BY 4.0 license.

The analytical methodology for *P. multocida*, *M. haemolytica*, and *H. somni* investigations is summarized in Figure 3.

We conducted all statistical analyses using Stata/MP 15.1 (Stata Corp, College Station, TX). Predictor variables, including bacterial culture results, were initially screened for potential associations with health status using Pearson’s chi-squared or Fisher’s exact tests. Variables associated with health status at *p* ≤ 0.20 were then evaluated in univariable mixed-effects logistic regression models (*xtlogit*), with herd included as a random effect.

Age, breed, and gender were considered as potential confounders based on biological plausibility and previous findings (31). Age as a categorical variable and breed were not evaluated further because of limited variation in the study population. Age as a continuous variable and gender were evaluated in univariable models. Although variation in gender was also limited, with 11 females and 77 males, it met the screening criterion of *p* ≤ 0.20 in univariable testing and was therefore assessed as a potential confounder by adding it separately to each model containing a bacterial predictor associated with health status at *p* ≤ 0.20. Gender did not notably change the bacterial effect estimates; therefore, no multivariable model was retained, and only univariable mixed-effects regression results for bacterial culture variables are presented. Associations with *p* < 0.05 were considered statistically significant.

To assess the performance of NS and NPS sampling sites, BAL was used as the reference test. Agreement between upper airway sites and BAL was evaluated using percent positive agreement (PPA), Cohen’s kappa, and McNemar’s exact test. PPA between two sites was calculated as the number of calves positive at both sites divided by the average number of calves positive at either site, multiplied by 100, as described by others (30). Kappa values were interpreted using the Landis and Koch scale: < 0.00 = poor; 0.00–0.20 = slight; 0.21–0.40 = fair; 0.41–0.60 = moderate; 0.61–0.80 = substantial; and 0.81–1.00 = almost perfect (33). McNemar’s exact test was applied to compare the marginal proportions of positive results between two sites (30). Positive and negative predictive values (PPV, NPV) were calculated relative to BAL, with PPV defined as the proportion of test positives that were true positives, and NPV as the proportion of test negatives that were true negatives.

All analyses were conducted using data from fattening calves, except for the descriptions of clinical findings and the calculations of agreement and predictive values of sampling sites in healthy and diseased calves, where data from the parallel dairy calf study (31) were included to increase statistical power.

#### 2.4.3 Sample size calculation

Post-hoc sample size calculations were performed to assess the achieved sample size for selected clinically relevant comparisons. These included the association between clinical disease and dominant, abundant growth of the most frequent pathogen in BAL, and agreement between upper and lower airway sites for detection of this pathogen. A corresponding calculation for upper airway detection was included to support interpretation. Other analyses, including those involving less frequent bacteria, low-prevalence findings, bacterial amount categories, and subgroups, should be considered exploratory.

Sample size was calculated to achieve 80% power at a 5% significance level. Due to difficulties in finding healthy fattening calves to sample, calculations were based on an allocation ratio of 1:2.5 (healthy: diseased). For NS or NPS samples, 43 healthy and 108 diseased calves were required to detect a 25-percentage point difference in the proportion of positive culture results, assuming a 60% prevalence of the most common pathogen in diseased calves. For BAL samples, 24 healthy and 58 diseased calves were required to detect a 30-percentage point difference between groups, assuming a 40% prevalence of dominant and abundant pathogen growth in diseased calves. For McNemar’s test, 117 calves with paired sampling sites were needed to detect a 15-percentage point difference, assuming 25% discordance in culture results.

## 3 Results

### 3.1 Clinical examination findings

**Figure 4:**
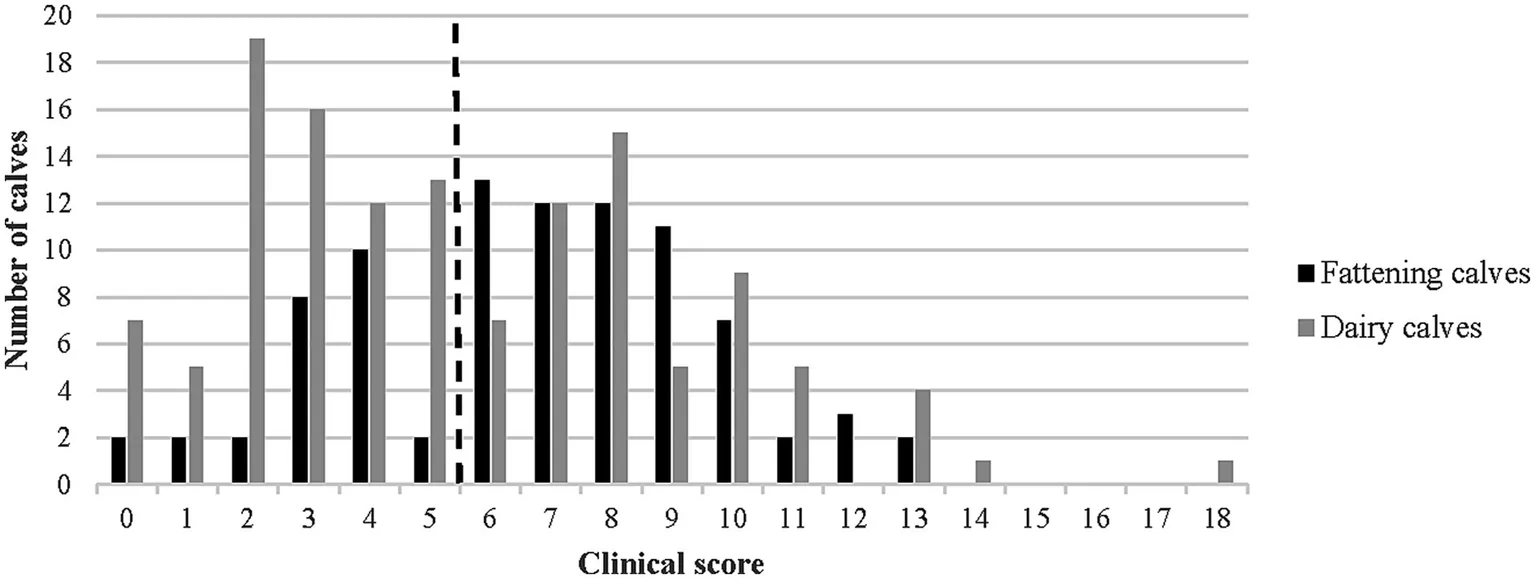
Distribution of total clinical scores (CS; range 0–18) among 88 fattening calves from seven herds and 131 dairy calves from nine herds. Clinical scoring was based on a chart modified from Klem et al. examining six parameters: respiratory rate, rectal temperature, cough, nasal discharge, lung auscultation, and general appearance, each scoring 0–3 points. Calves with a total CS > 5 were classified as diseased; the cut-off is shown by the vertical dotted line.

The distribution of total clinical scores (CS) is shown in Figure 4. Dairy calves from a parallel study are also described in this figure due to their inclusion in the assessment of agreement between upper and lower airway sampling sites in healthy vs. diseased calves (last part of results section).

Among fattening calves, 26 were classified as healthy and 62 as diseased, while among dairy calves, 72 were healthy and 59 were diseased. The median CS for all calves was 6 (range: 0–18); with medians of 7 (range: 0–13) in fattening and 5 (range: 0–18) in dairy calves. Clinical score values were evenly distributed around the cut-off value between healthy and diseased calves.

Respiratory signs were common in both populations (Table 1). The most frequent findings were nasal discharge (fattening: 84% [74/88]; dairy: 79% [103/131]), abnormal auscultation findings (fattening: 80% [70/88]; dairy: 51% [67/131]), and cough (fattening: 66% [58/88]; dairy: 56% [74/131]). Less frequent signs were elevated rectal temperature (fattening: 28% [25/88]; dairy: 13% [17/131]) and altered general appearance (fattening: 16% [14/88]; dairy: 24% [31/131]).

### 3.2 Occurrence of BRD-associated pathogens in fattening calves

Overall, *Pasteurella multocida* and *Mannheimia haemolytica* were the most frequently detected bacteria across sampling sites, with *P. multocida* predominating in bronchoalveolar lavage samples.

#### 3.2.1 Cultured material

Nasal and nasopharyngeal swabs (NS and NPS) from 88 fattening calves, and bronchoalveolar lavage (BAL) samples from 86, were obtained for culture. For the BAL procedure, all except five calves received 60 mL fluid; those five required two 60 mL attempts for successful fluid retrieval. The mean fluid return was 31% ± 12% (range: 10%–57%), with minimal variation across culture result outcomes (Supplementary Table S5). Twenty-five BAL cultures were bacteria-negative and followed up by culturing the corresponding enriched BAL samples.

#### 3.2.2 *Pasteurellaceae* species

Detection rates of *Pasteurellaceae* spp. at the herd, calf, and sampling site levels are summarized in Table 2.

**Table 2:** Detection of *Pasteurellaceae* species at the herd, calf, and sampling site levels, based on culture results from 88 calves across seven fattening herds.

Culture result	Percentages (*n*) of herds and calves with each culture result					
	Herd (*n* = 7)	Calf (*n* = 88)	NS (*n* = 88)	NPS (*n* = 88)	BAL (*n* = 86)	Enriched BAL^2^ (*n* = 25)
Species, detected^1^						
*P. multocida*	100% (7)	76% (67)	43% (38)	47% (41)	55% (47)	24% (6)
*M. haemolytica*	100% (7)	81% (71)	68% (60)	59% (52)	27% (23)	12% (3)
*H. somni*	57% (4)	31% (27)	15% (13)	19% (17)	15% (13)	0% (0)
No *Pasteurellaceae* spp.	0% (0)	6% (5)	11% (10)	17% (15)	34% (29)	68% (17)
Combinations, detected^3^^,^^4^						
*P. multocida + M. haemolytica*	43% (3)	40% (35)	20% (18)	19% (17)	12% (10)	4% (1)
*P. multocida + H. somni*	0% (0)	2% (2)	2% (2)	3% (3)	5% (4)	0% (0)
*M. haemolytica + H. somni*	0% (0)	6% (5)	8% (7)	8% (7)	5% (4)	0% (0)
*P. multocida + M. haemolytica + H. somni*	57% (4)	23% (20)	3% (3)	6% (5)	5% (4)	0% (0)
No *Pasteurellaceae* spp. combination	0% (0)	30% (26)	66% (58)	64% (56)	74% (64)	96% (24)

NS, nasal swab; NPS, nasopharyngeal swab; BAL, bronchoalveolar lavage.

1 For sole species: Herds were considered positive for a given species if ≥ 1 calf in the herd was positive; and calves were positive if ≥ 1 sampling site (NS, NPS, or BAL, including enriched samples) was positive.

2 Enriched BAL: BAL fluid enriched with BHI broth following bacteria negative culture from the corresponding non-enriched BAL fluid, providing additional lower airway isolates.

3 For species combinations: Herds were positive if the species involved were present in the herd; and calves were positive if the combination occurred within the calf (possibly from different sampling sites).

4 Herds, calves, and sampling sites, were assigned to maximum one combination category (e.g., a herd where all three species occurred counted only in the three-species category, not also in the two-species categories).

**Figure 5:**
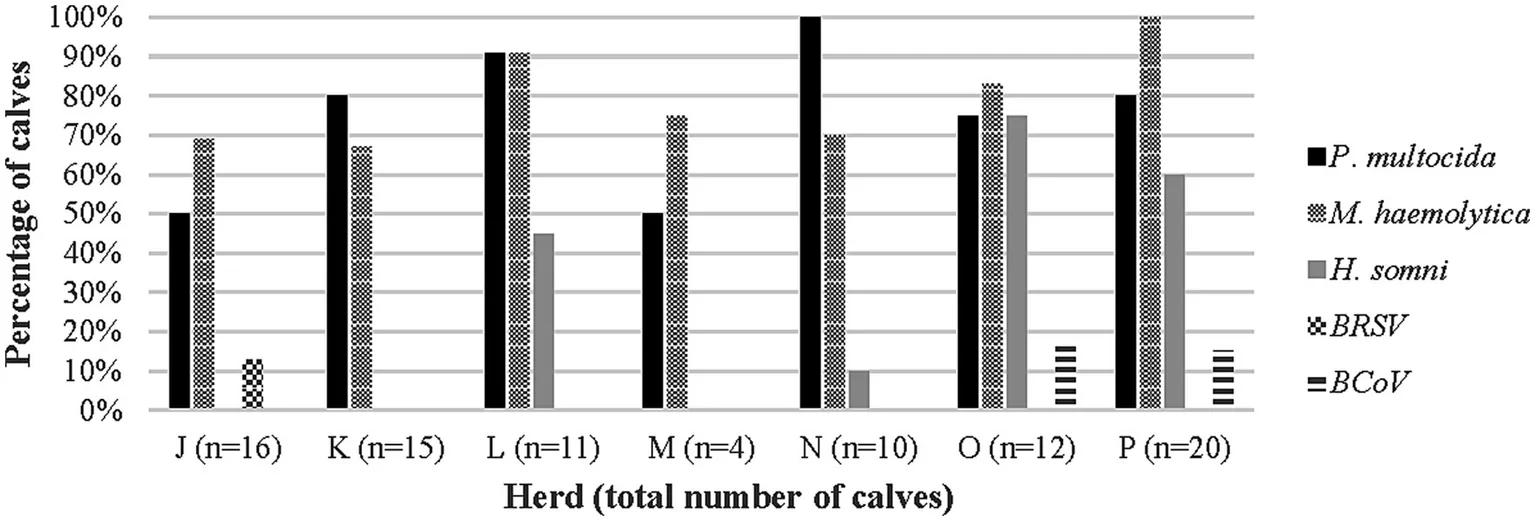
The percentage of calves per herd with BRD-associated pathogens detected in nasal swabs, nasopharyngeal swabs, or bronchoalveolar lavage samples among 88 calves in seven fattening herds.

In all herds, *P. multocida* and *M. haemolytica* were detected and were the most prevalent pathogens (Figure 5). *Histophilus somni* was found in four herds, occurring at similar frequencies to the other two *Pasteurellaceae* spp. in two of them. At the calf level, 94% (83/88) of calves were positive for *Pasteurellaceae* spp. (≥ 1 sampling site positive), with *M. haemolytica* being the most frequently identified species, closely followed by *P. multocida* and then *H. somni* (Table 2). In 70% (62/88) of calves, ≥ 2 *Pasteurellaceae* spp. were isolated.

At the sampling site level, the majority of calves were positive for *Pasteurellaceae* spp., with detection rates of 89% (78/88) in NS, 83% (73/88) in NPS, and 66% (57/86) in BAL samples. *M. haemolytica* was the most frequently detected species in the upper airway sampling sites, followed by *P. multocida* and *H. somni.* From upper to lower airway sampling sites, isolation rates increased for *P. multocida*, decreased for *M. haemolytica,* and remained similar for *H. somni*. Of the *Pasteurellaceae* spp. positive BAL cultures, *P. multocida* was detected in 82% (47/57), *M. haemolytica* in 40% (23/57), and *H. somni* in 23% (13/57). Among these BAL cultures, co-occurrence with at least one other *Pasteurellaceae* sp. was observed in 38% (18/47) of *P. multocida*-positive cultures, 78% (18/23) of *M. haemolytica*-positive cultures, and 92% (12/13) of *H. somni*-positive cultures. Across all sampling sites, co-occurring *Pasteurellaceae* spp. were found in 34% (30/88) of NS, 36% (32/88) of NPS, and 26% (22/86) of BAL, with *P. multocida* and *M. haemolytica* being the most common combination.

#### 3.2.3 Other BRD-associated pathogens

From the 88 calves, *Trueperella pyogenes* and *Bibersteinia trehalosi* were not detected in any of the NS, NPS, or BAL samples. Serum from 85 out of 88 calves (three samples missing) were analyzed for antibodies against *M. bovis*, and all tested negative, with no doubtful samples. Viral examinations of NS samples from all 88 calves revealed two calves from one herd testing positive for BRSV; while two calves from a second herd and three calves from a third herd tested positive for BCoV (Figure 5). All calves tested negative for BPIV3, BVDV, and BHV1.

#### 3.2.4 Impact of upper airway contamination

Of 57 *Pasteurellaceae* spp. positive BAL cultures, potential upper airway contamination (according to the definition described previously) was found in 14% (8/57) of samples, from which three *P. multocida*, four *M. haemolytica*, and four *H. somni* isolates derived. Among 29 *Pasteurellaceae* spp. negative BAL cultures, 97% (28/29) had no growth or < 3 individual colonies of contaminants, while the remaining 3% (1/29) had 5–10 individual colonies of contaminants.

### 3.3 Antimicrobial resistance (AMR) pattern of *Pasteurellaceae* in calves from fattening herds

Antimicrobial resistance levels were generally low among *Pasteurellaceae* spp., with the main exceptions being penicillin resistance in *M. haemolytica* and streptomycin resistance in all three *Pasteurellaceae* spp.

The phenotypic AMR pattern of 132 *P. multocida*, 138 *M. haemolytica*, and 43 *H. somni* isolates from the 88 fattening calves were investigated. Among these isolates, no resistance was observed to amoxicillin-clavulanic acid, trimethoprim sulfamethoxazole, enrofloxacin, or florfenicol. Tetracycline resistance was detected in one *P. multocida* isolate (1/132). All *P. multocida* and *H. somni* isolates were susceptible to penicillin, while 14% (20/138) of *M. haemolytica* were penicillin-resistant, identified in both upper (n = 16) and lower (n = 4) airway sampling sites. For streptomycin, 57% (75/132) of *P. multocida* isolates were susceptible, 9% (12/132) susceptible at increased exposure, and 34% (45/132) were resistant. Corresponding numbers for *M. haemolytica* isolates were 74% (102/138), 4% (6/138), and 22% (30/138); and for *H. somni* isolates, 58% (25/43), 26% (11/43), and 16% (7/43), respectively.

At the calf level, penicillin-resistant *M. haemolytica* were detected in 18% (13/71) of calves positive for *M. haemolytica*, originating from five herds (J, L, M, N, and P). Streptomycin-resistant isolates of each species were identified in 37% (25/67) of *P. multocida*-positive calves (five herds: J, K, L, N, P), 24% (17/71) of *M. haemolytica*-positive calves (five herds: J, L, M, N, P), and 26% (7/27) of *H. somni*-positive calves, all from one herd (P).

### 3.4 Culture result interpretation in calves from fattening herds

Among investigated pathogens, detection of *P. multocida* in BAL showed the clearest difference between healthy and diseased calves.

#### 3.4.1 Culture results in healthy vs. diseased calves

At the calf level, *P. multocida* was detected in 58% (15/26) of healthy and 84% (52/62) of diseased calves, *M. haemolytica* in 69% (18/26) and 85% (53/62), and *H. somni* in 12% (3/26) and 39% (24/62), respectively. At the sampling site level (Table 3), isolation rates of *M. haemolytica* were generally higher than those of *P. multocida* in NS and NPS for both healthy and diseased calves. In BAL, however, *P. multocida* was the predominant species in diseased calves, while isolation rates were similar for *P. multocida* and *M. haemolytica* in healthy calves. Across all sampling sites, *H. somni* was consistently more common in diseased calves. Detailed within-herd distributions of *Pasteurellaceae* spp. at both the calf and sampling site levels are provided in Supplementary Table S6.

**Table 3:** Semi-quantitative culture results for *Pasteurellaceae* species in sampling sites of 26 healthy and 62 diseased calves from seven fattening herds, and their association with health status based on Pearson’s chi-squared test (≥5 observations per health group) or Fisher’s exact test (<5 observations in one or both groups).

Culture result		Nasal swab % (*n*)			Nasopharyngeal swab % (*n*)			Bronchoalveolar lavage % (*n*)		
Species	Gradient^1^	Total (*n* = 88)	Healthy (*n* = 26)	Diseased (*n* = 62)	Total (*n* = 88)	Healthy (*n* = 26)	Diseased (*n* = 62)	Total (*n* = 86)	Healthy (*n* = 26)	Diseased (*n* = 60)
*P. multocida*	Detected	43% (38)	42% (11)	44% (27)	47% (41)	42% (11)	48% (30)	55% (47)	23% (6)	68% (41) ***
	Dominant	24% (21)	31% (8)	21% (13)	27% (24)	23% (6)	29% (18)	48% (41)	12% (3)	63% (38) ***
	-Pure^2^	1% (1)	0% (0)	2% (1)	7% (6)	4% (1)	8% (5)	34% (29)	12% (3)	43% (26) ***
	Dominant + abundant	20% (18)	23% (6)	19% (12)	10% (9)	8% (2)	11% (7)	31% (27)^3^	8% (2)	42% (25) ***
*M. haemolytica*	Detected	68% (60)	46% (12)	77% (48) ***	59% (52)	58% (15)	60% (37)	27% (23)	19% (5)	30% (18)
	Dominant	45% (40)	27% (7)	53% (33) ***	32% (28)	23% (6)	35% (22)	10% (9)	8% (2)	12% (7)
	-Pure^2^	0% (0)	0% (0)	0% (0)	2% (2)	4% (1)	2% (1)	5% (4)	8% (2)	3% (2)
	Dominant + abundant	43% (38)	27% (7)	50% (31) ***	18% (16)	4% (1)	24% (15) ***	3% (3)	0% (0)	5% (3)
*H. somni*	Detected	15% (13)	4% (1)	19% (12) **	19% (17)	12% (3)	23% (14)	15% (13)	4% (1)	20% (12) **
	Dominant	5% (4)	4% (1)	5% (3)	5% (4)	8% (2)	3% (2)	7% (6)	0% (0)	10% (6) *
	-Pure^2^	0% (0)	0% (0)	0% (0)	0% (0)	0% (0)	0% (0)	0% (0)	0% (0)	0% (0)
	Dominant + abundant	5% (4)	4% (1)	5% (3)	1% (1)	0% (0)	2% (1)	5% (4)	0% (0)	7% (4)
No *Pasteurellaceae* spp.	Detected	11% (10)	35% (9)	2% (1)	17% (15)	23% (6)	15% (9)	34% (29)	62% (16)	22% (13)
*P. multocida + M. haemolytica*	Detected	20% (18)	23% (6)	19% (12)	19% (17)	19% (5)	19% (12)	12% (10)	4% (1)	15% (9)
*P. multocida + H. somni*	Detected	2% (2)	4% (1)	2% (1)	3% (3)	4% (1)	3% (2)	5% (4)	0% (0)	7% (4)
*M. haemolytica + H. somni*	Detected	8% (7)	0% (0)	11% (7) **	8% (7)	4% (1)	10% (6)	5% (4)	4% (1)	5% (3)
*P. multocida + M. haemolytica + H. somni*	Detected	3% (3)	0% (0)	5% (3)	6% (5)	4% (1)	6% (4)	5% (4)	0% (0)	7% (4)

*p*-values are indicated as follows: no annotation = *p* > 0.20; *p ≤ 0.20; **p ≤ 0.10; ***p < 0.05.

1 Detected = all positive cultures; dominant = all dominant and pure cultures; dominant + abundant = dominant with growth in > half of 1st dilution of culture (nasal swab, nasopharyngeal swab) or ≥ 10 colonies (bronchoalveolar lavage [BAL]).

2 Pure cultures were included in the “dominant” category for statistical analyses but were also analyzed separately if observed in ≥ 5 calves.

3 Out of BAL cultures with dominant, abundant growth of *P. multocida*, 74% (20/27) were pure cultures.

Initial statistical testing (Chi-squared or Fisher’s exact test) showed that the following culture result categories were associated with disease: all amount categories of *P. multocida-*positive BAL cultures; all categories of *M. haemolytica* in NS cultures; and dominant and abundant *M. haemolytica* cultures from NPS.

#### 3.4.2 Dominant cultures across species and sampling sites

Frequencies of dominant cultures differed between species and sampling sites. The proportion of positive cultures that were classified as dominant increased for *P. multocida* from upper to lower airway sites, comprising 55% (21/38) in NS, 59% (24/41) in NPS, and 87% (41/47) in BAL. The opposite trend was observed for *M. haemolytica*, with dominant cultures accounting for 67% (40/60), 54% (28/52), and 39% (9/23) of positive NS, NPS, and BAL cultures. *H. somni* was dominant in 31% (4/13), 24% (4/17), and 46% (6/13) of positive NS, NPS, and BAL cultures, respectively. Out of cultures where both *P. multocida* and *M. haemolytica* occurred, with or without the presence of *H. somni*, *P. multocida* was dominant in 48% (10/21), 36% (8/22), and 86% (12/14) of NS, NPS, and BAL cultures, respectively, while *M. haemolytica* was dominant in 52% (11/21), 32% (7/22), and 29% (4/14).

#### 3.4.3 Associations between health status and predictor variables by logistic regression

In univariable logistic regression analyses, *P. multocida* in BAL was the only bacterial finding consistently associated with clinical disease.

An overview of all variables tested in univariable models is provided in Supplementary Table S7. Univariable modeling identified five variables associated with disease: all amount categories of *P. multocida* cultures in BAL (detected, dominant, and dominant and abundant cultures; and pure cultures as a stratified category), and *M. haemolytica* detected in NS. Calves with *P. multocida* detected in BAL had 7-fold higher odds of clinical disease than those with negative cultures (OR: 6.75; 95% CI: 2.14–21.37; *p* < 0.01); most of these positive cultures (87%, 41/47) were dominant. The odds of disease were further elevated in calves with BAL cultures in which *P. multocida* was dominant (OR: 15.36; 95% CI: 3.37–70.13; *p* < 0.01) or both dominant and abundant (OR: 8.85; 95% CI: 1.78–44.10; *p* < 0.01).

Because *P. multocida* in BAL was strongly associated with disease, the association between *M. haemolytica* in NS and disease was further investigated after accounting for co-occurrence with *P. multocida* in BAL. Among 86 BAL-sampled calves, 58 were positive for *M. haemolytica* in NS; and of these, 64% (37/58) were also positive for *P. multocida* in BAL (Chi^2^, *p <* 0.02). After excluding the 37 calves that were positive for *M. haemolytica* in NS and positive for *P. multocida* in BAL, 49 BAL-sampled calves remained for analysis. In these calves, *M. haemolytica* in NS was detected in 36% (8/22) of healthy and 48% (13/27) of diseased calves, with no longer a significant association with disease (Chi^2^, *p =* 0.41).

### 3.5 Comparison of culture results in upper relative to lower airway sites

Agreement between upper airway sampling sites and BAL was generally slight to fair at the individual calf level.

#### 3.5.1 Detection of *Pasteurellaceae* species in NS and NPS relative to BAL in fattening calves

Agreement between upper and lower airway sampling sites for detecting *P. multocida*, *M. haemolytica*, and *H. somni*, as measured by kappa, ranged from slight to fair (Table 4). Among the three species, *P. multocida* in NPS showed the highest percent positive agreement (PPA) with BAL (67%), while PPA for other species across sites did not exceed 58%. For *M. haemolytica*, positive results were significantly more frequent in NS and NPS than in BAL. Positive predictive values (PPV) of NS and NPS relative to BAL were higher for *P. multocida* than for the other two species, while negative predictive values were lower. Between the two upper airway sites, NPS agreed better with BAL than NS for *P. multocida* and, to a lesser extent, *M. haemolytica*, whereas NS performed better than NPS for *H. somni.*

**Table 4:** Agreement and predictive values of NS and NPS culture results relative to BAL for detection of *Pasteurellaceae* species in 86 calves from seven fattening herds.

Population of calves	Health status	Species	Upper airway site	Calves (*n*) per combination (BAL/upper airway site)				Percent positive agreement (95% CI)^a^	Kappa (95% CI)	Classification	*p*^b^	PPV %	NPV %
				+/+	+/−	−/+	−/−						
Fattening	Total (*n* = 86)	*P. multocida*	NS	23	24	15	24	54 (44–65)	0.10 (0.00–0.21)	Slight	0.20	61	50
			NPS	29	18	11	28	67 (57–77)	0.33 (0.22–0.44)	Fair	0.26	73	61
		*M. haemolytica*	NS	21	2	37	26	52 (41–63)	0.22 (0.14–0.30)	Fair	0.00	36	93
			NPS	21	2	29	34	58 (46–69)	0.33 (0.24–0.42)	Fair	0.00	42	94
		*H. somni*	NS	6	7	7	66	46 (27–65)	0.37 (0.26–0.47)	Fair	1.00	46	90
			NPS	4	9	12	61	28 (11–44)	0.13 (0.02–0.24)	Slight	0.66	25	87
Fattening and dairy	Total (*n* = 199)	*P. multocida*	NS	47	30	44	78	56 (49–64)	0.24 (0.17–0.31)	Fair	0.13	52	72
			NPS	52	25	36	86	63 (56–70)	0.37 (0.30–0.44)	Fair	0.20	59	77
	Healthy (*n* = 92)	*P. multocida*	NS	17	5	22	48	56 (43–68)	0.36 (0.27–0.46)	Fair	0.00	44	91
			NPS	17	5	19	51	59 (46–71)	0.41 (0.31–0.51)	Moderate	0.01	47	91
	Sick (*n* = 107)	*P. multocida*	NS	30	25	22	30	56 (47–66)	0.12 (0.03–0.22)	Slight	0.77	58	55
			NPS	35	20	17	35	65 (56–74)	0.31 (0.21–0.41)	Fair	0.74	67	64

The table also presents results for detection of *P. multocida* in healthy and diseased fattening and dairy calves combined, including 26 healthy and 60 diseased calves from seven fattening herds, and 66 healthy and 47 diseased calves from nine dairy herds.

NS, nasal swab; NPS, nasopharyngeal swab; BAL, bronchoalveolar lavage; PPV, positive predictive value; NPV, negative predictive value.

a The 95% confidence interval (CI) was calculated using the Wald (normal approximation) method.

b *p*-value is based on McNemar’s exact test.

#### 3.5.2 Detection of *P. multocida* in NS and NPS relative to BAL in healthy and diseased fattening and dairy calves

Agreement between upper and lower airway sampling sites for *P. multocida* detection, as measured by kappa, varied with health status, with fair to moderate agreement in healthy calves and slight to fair in diseased calves (Table 4). For NS and NPS relative to BAL, percent positive agreement was similar between healthy and diseased calves, while PPVs were higher in diseased calves (PPV ≤ 67%) than in healthy calves (PPV ≤ 47%). In healthy calves, positive results were significantly more frequent in NS and NPS than in BAL, whereas in diseased calves, where BAL detection was much more frequent, this difference was not observed. In diseased calves, NPS performed better than NS for both kappa and PPA, while in healthy calves, NS and NPS showed comparable performance.

#### 3.5.3 *P. multocida*-positive cultures in NS and NPS relative to BAL at the group level

Despite limited agreement at the individual level, group-level isolation rates for *P. multocida* were similar across sampling sites in diseased calves.

**Figure 6:**
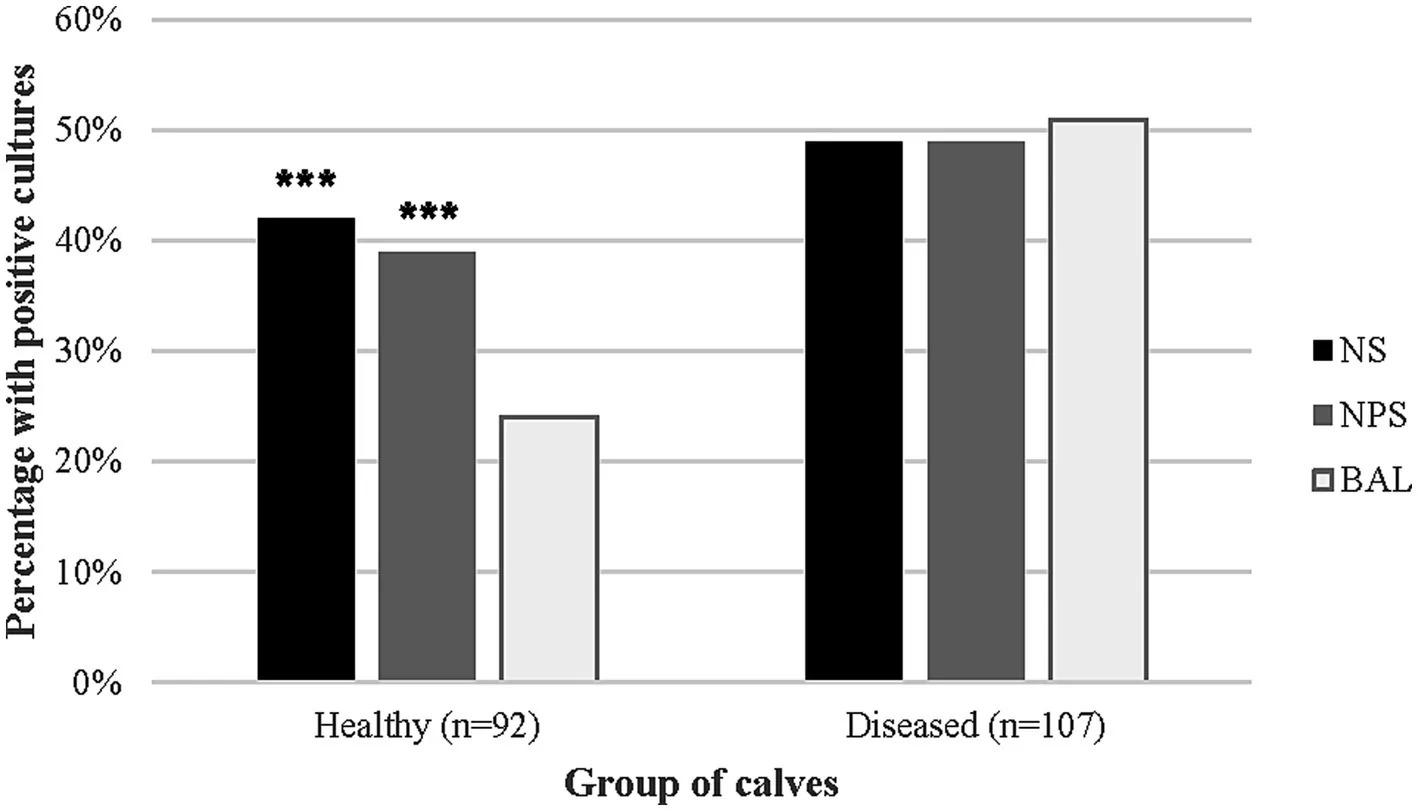
Proportions of healthy and diseased calves with *P. multocida*-positive cultures from three sampling sites. Cultures were obtained from NS, NPS, and BAL samples from 92 healthy and 107 diseased calves, including 26 healthy and 60 diseased calves from seven fattening herds, and 66 healthy and 47 diseased calves from nine dairy herds. NS, nasal swab; NPS, nasopharyngeal swab; BAL, bronchoalveolar lavage. ***p < 0.05 based on McNemar’s exact test using BAL as reference test for NS and NPS.

When culture results were aggregated at the group level (Figure 6), healthy calves showed significantly higher proportions of *P. multocida*-positive cultures in NS and NPS than in BAL (approximately 1.6–1.8 times higher; *p* < 0.05), whereas diseased calves had similar positive proportions across all three sites. NS and NPS showed comparable proportions of *P. multocida*-positive cultures within both health groups.

At the herd level (Supplementary Table S8), both the healthy and diseased group in fattening herds, and the healthy group in dairy herds, generally followed the respective group-level pattern shown in Figure 6, to varying degrees. The diseased group in dairy herds, however, resembled more the healthy group-level pattern.

## 4 Discussion

### 4.1 Occurrence of BRD-associated pathogens

In this study, *P. multocida* was the most frequently isolated BRD-associated bacterium in samples from the lower airways, while *M. haemolytica* predominated in the upper airway sampling sites. Traditionally, *M. haemolytica* has been considered the primary bacterial cause of BRD in transported calves raised for beef production (34–36). However, studies in multiple regions over the past few decades have challenged this view, reporting *P. multocida* to be the most common *Pasteurellaceae* sp. from lower airway samples of such calves (37–41). It has been suggested that this potential shift from *M. haemolytica* to *P. multocida* as the dominant pathogen could be attributed to changes in virulence among these bacteria, effective AMs, development of AMR (39), and/or the broader availability of effective vaccines against *M. haemolytica* (37, 42). In Norway, BRD vaccination has only recently become more common (43). Although some study herds did use *M. haemolytica* vaccines, overall vaccination rates are much lower than in many other countries, so any vaccine effect is likely limited. Moreover, AM use in Scandinavia is traditionally prudent (44), with low levels of AMR (45–47). Together, this suggests that the predominance of *P. multocida* in this study is unlikely to be primarily driven by vaccine- or AM selection pressure and may instead indicate persistent circulation of clinically relevant *P. multocida* strains. This is consistent with diagnostic laboratory experience at NMBU, where *P. multocida* has been consistently detected in respiratory samples from clinical BRD cases over the past three decades (anecdotal observation).

*H. somni* was less frequently isolated in this study and not detected in all herds. A similar pattern was observed in a Swiss study, where isolation was infrequent (20), and in a Belgian study, where the pathogen was not detected (48). In contrast, studies from the USA and Italy have reported more frequent isolation of *H. somni* (35, 49). These findings support that its occurrence may vary with environment and production system, perhaps with lower occurrence in smaller-scale systems such as Norwegian fattening herd operations (20). Low detection rates of *H. somni* may also partly reflect the use of culture-based detection, which can underestimate its true occurrence because the bacterium is slow-growing and easily overgrown by commensal flora (23). However, culture-positive findings are likely to be more clinically relevant than PCR-based detection, which may detect small amounts of DNA from both live and dead bacteria.

The absence of serum antibodies to *M. bovis* aligns with the current understanding that this pathogen is not present in Norway. Although the sample size was small, the herds included had high-risk traits that may be typical of *M. bovis* positive herds elsewhere, including chronic pneumonia issues (50) and frequent calf purchases from multiple farms (51). Given the presence of *M. bovis* in neighboring countries (13, 14) and the emergence of a new clone in Northern Europe (15), continued vigilance and surveillance remain important.

Viral respiratory pathogens were infrequently detected, which was expected given the selection of herds with chronic respiratory problems rather than acute, typically viral outbreaks. Clinical findings further suggest that most calves were in a chronic or late stage of BRD, with relatively few showing signs of fever or depression. Since viral shedding usually occurs during the acute stage of disease (32), the one-time sampling at a random time may have missed this phase and underestimated viral presence and relevance. The absence of BVDV and BHV1 was also expected, as Norway is considered free from these viruses at the national level (52, 53). Overall, the low occurrence of several respiratory viruses and *M. bovis* reduce potential sources of bias and strengthen interpretations regarding the clinical relevance of *Pasteurellaceae* spp.

### 4.2 Antimicrobial resistance pattern of *Pasteurellaceae* species

Antimicrobial resistance to BRD AMs was rare among *Pasteurellaceae* spp. in this study. The only resistance detected was penicillin resistance in *M. haemolytica*, streptomycin resistance in all three *Pasteurellaceae* spp., and tetracycline resistance in one *P. multocida* isolate.

The overall low levels of penicillin resistance among *Pasteurellaceae* spp. in this study were similar to findings from other Scandinavian reports (25, 31, 45), likely resulting from a tradition of prudent AM use (44) and low occurrence of *M. bovis* (11, 13), allowing the use of penicillin as a primary choice for BRD treatment. Low levels of penicillin resistance are also reported for *P. multocida* and *M. haemolytica* outside Scandinavia (7, 37, 46), although higher levels occur in some regions (20, 54). This regional variability may reflect differences in AM use, herd size, production system, pathogen occurrence, and management. Larger herds in intensive systems, such as feedlots and veal calf systems, may have higher BRD pressure and more frequent use of group treatments with broad-spectrum AMs (7, 55), several of which are available as single-dose formulations. In contrast, smaller herds typical of Norwegian production systems may allow closer individual monitoring and make daily treatment with common narrow-spectrum penicillin formulations more feasible (44), thereby limiting selection pressure.

Nevertheless, penicillin resistance in *M. haemolytica* was higher in the present fattening calves than previously reported in Norwegian dairy calves (31), possibly reflecting higher disease pressure and AM exposure in fattening herds (7). This difference was not observed for *P. multocida*, as no penicillin resistance was detected in either study.

For other AMs, several studies report low florfenicol resistance and more frequent tetracycline resistance (7, 20, 37, 54). Our findings were similar for florfenicol, but differed for tetracycline, with resistance detected in only one isolate. Streptomycin resistance among *Pasteurellaceae* spp. was common, but this finding has less direct relevance for current BRD treatment recommendations. Streptomycin resistance among *Pasteurellaceae* spp. is well documented and associated with plasmid-borne genes, often clustered with other AMR genes (56). These clusters are likely maintained by streptomycin use or co-selection from other AMs.

The overall low occurrence of AMR in fattening herds with high BRD pressure and previous AM use supports the assumptions underlying national AM therapy guidelines, namely that the most relevant BRD-associated bacteria are penicillin-susceptible. However, the detection of penicillin-resistant *M. haemolytica* highlights the continued importance of AM susceptibility testing, even in regions with generally low AMR levels. Regional variability in resistance further underscores the importance of local knowledge of respiratory pathogens and their AMR patterns, which may support the use of narrow-spectrum penicillin to limit selection pressure.

### 4.3 Culture results associated with respiratory disease

The strongest association with clinical disease was observed for *P. multocida* detected in BAL samples, across all semi-quantitative culture categories. Most *P. multocida*-positive BAL cultures were dominant, and more than half occurred without co-occurring *Pasteurellaceae* spp. When other *Pasteurellaceae* spp. were present, they were usually non-dominant. Together with the achieved power for this analysis, these findings support an association between clinical disease and *P. multocida* in BAL.

This finding is consistent with previous studies reporting associations between isolation of *P. multocida* in lower airway samples and morbidity (22) or increased concentrations of acute phase proteins (57). However, other studies have found no difference in lower airway detection rates of *P. multocida* between healthy and diseased calves (25, 27), showing how clinical relevance of pathogens may differ from one population to another. In Norwegian dairy calves, *P. multocida* in BAL cultures was associated with BRD only if present with dominant and abundant (≥ 10 colonies) growth (31), suggesting that lower bacterial amounts may be less clinically relevant. In the present study, many diseased calves showed dominant, but not necessarily abundant growth of *P. multocida*. This could reflect chronic or resolving disease, prior AM treatment, or clinical signs attributable to other causes. The higher odds ratios observed for dominant and abundant categories compared with detection alone may suggest that bacterial amount has clinical relevance. However, broad confidence intervals, semi-quantitative data, and the cross-sectional study design limit firm conclusions. Because sampling was performed only once, disease stage was unknown, and it was not possible to determine whether detected bacteria represented primary causative agents, secondary infections, or commensal colonization. Therefore, the observed associations should not be interpreted as evidence of causation.

The statistical modeling was also limited by the size and structure of the dataset. Several bacterial culture variables across sampling sites involved sparse observations or co-occurring bacterial findings. When statistically significant findings were identified, these were mainly related to *P. multocida* in BAL, such as the association between *M. haemolytica* detected in NS and disease, which likely reflected co-occurrence with *P. multocida* in BAL rather than an independent association.

Overall, the strong association between clinical disease and *P. multocida* in BAL, suggests that *P. multocida* was the most clinically relevant pathogen among investigated bacteria, and should be considered an important target in diagnostic investigations and control strategies for Norwegian fattening herds with enzootic pneumonia.

### 4.4 Comparison of culture results from upper and lower airway sampling sites

At the individual level, agreement between upper airway (NS or NPS) cultures and BAL for pathogen detection was generally slight to fair, indicating that upper airway cultures did not reliably reflect bacterial presence in the lungs. For *M. haemolytica*, and for *P. multocida* in healthy calves, a significantly higher proportion of calves were positive in NS and NPS than in BAL, suggesting that upper airway sites may overestimate bacterial presence in the lungs in some cases. Positive upper airway cultures for P. multocida were more predictive of positive BAL cultures in diseased than in healthy calves, as shown by the higher PPVs, likely reflecting the higher pathogen prevalence in the lungs of clinically affected animals.

These findings align with several previous studies reporting slight to moderate agreement (22, 23, 27, 28) for pathogen detection between upper and lower airway sites, and better performance in diseased calves (27). However, substantial to almost perfect (29, 30) agreement has also been reported. As agreement is highly influenced by lung pathogen prevalence (58), variation across studies is likely due to differences in study populations, disease stage, and timing of sampling. In fattening calves, for instance, lung pathogen prevalence appears to be higher earlier in the feeding period, when animals are exposed to multiple stressors, than closer to slaughter (22, 59). Differences in lower airway sampling methods, such as the use of BAL, which samples a single lobe, rather than transtracheal wash, which might better represent the entire lung (17, 30), may also contribute to reduced concordance between studies. Most calves in this study showed signs consistent with chronic or late-stage BRD. Under these conditions, bacteria may have already been cleared from the lungs of some diseased animals or become more localized, potentially reducing agreement.

Because individual-level agreement was limited, we also evaluated whether upper and lower airway sites showed better concordance at the group level for isolation of *P. multocida*. Isolation rates were similar across NS, NPS, and BAL in diseased calves, particularly in fattening herds, while in healthy calves, the proportions of positive cultures were significantly higher in NS and NPS than in BAL. These findings suggest that, at the group level, NS or NPS may reasonably well reflect bacterial presence in the lower airways, provided all sampled calves have clinical signs of pneumonia and enough calves are tested. Our group-level interpretation aligns with previous studies reporting improved concordance between upper and lower airway sites at the group level (22, 30, 60). For instance, one study in feedlot calves reported only moderate agreement between NPS and BAL for detecting *P. multocida* at the individual level, but similar isolation rates at the group level (22). Moreover, previous studies have found *P. multocida* isolates to be genetically identical in 70% of paired NS and transtracheal swabs (61), and 75% of paired NPS and tracheobronchial lavage samples (27), supporting the potential value of upper airway sampling for AM susceptibility testing at the group level, if a sufficient number of calves are sampled.

The diagnostic implications therefore differ between individual calves and groups. For individual calves, NS and NPS cultures should not be considered accurate indicators of lower airway bacterial status; BAL is likely more reliable. At the herd level, however, upper airway sampling may provide a practical, less invasive alternative for identifying clinically relevant bacteria and obtaining isolates for AM susceptibility testing in calves with clinical signs of BRD. This is relevant because BRD is primarily a herd-level problem, and AM treatment decisions are typically made for groups of calves.

A final observation regarding the value of upper airway sampling was that, at the individual level, NPS performed moderately better than NS, particularly for detection of *P. multocida* in diseased calves. This is consistent with previous studies suggesting that the nasopharyngeal microbiota more closely resembles the lower airway microbiota (17, 62), but contrasts with studies in dairy calves that have found similar performance between the two sampling sites (30, 31). At the group level, isolation rates were similar between NS and NPS. Because NPS is more expensive and technically demanding than NS, its moderate individual-level benefit may not outweigh the additional effort and cost compared with NS, especially when sampling is conducted for herd-level assessment.

### 4.5 Limitations

When evaluating the internal validity of this study, referring to the representativeness of the results for the source population and the impact of systematic biases (58), several limitations should be considered. First, the cross-sectional design precludes conclusions regarding causality, and pathogen detection must therefore be interpreted as associative rather than causal. Second is the potential for misclassification bias. Calves were classified as healthy or diseased based on clinical scoring. Severely ill calves were excluded and many had scores near the cut-off, making results sensitive to the threshold. All examinations were done by the same experienced veterinarian to reduce variability and misclassification, and we consider most classifications to be as accurate as reasonably possible. Nonetheless, some calves may have been at an intermediate stage and not clearly healthy or diseased. In addition, since health status was determined by a single clinical examination, some calves classified as healthy may have had subclinical infections (63). A third limitation is the sample size. Power was limited for analyses with fewer calves than the calculated sample size and bacterial categories with sparse observations. As a result, existing associations with disease for certain pathogens may have gone undetected. A forth limitation is the use of blind BAL as an imperfect reference test. BAL typically samples a single, random lung lobe and, in sedated calves, may systematically sample less affected diaphragmatic lobes (64). Therefore, focal or unevenly distributed infections may have been missed, leading to some false negative results and reduced agreement between sampling sites. Negative BAL results in diseased calves could also reflect viral infections, though viral occurrence appeared low, or persistent lesions without residual bacteria, or AM treatment prior to the 14 day exclusion window. In addition, nasopharyngeal passage of the BAL tube introduces a risk of contamination. However, the low number of contaminated samples, consistent with previous reports (23, 31), suggests minimal impact. A fifth limitation is the AM susceptibility testing of only one isolate per species per sampling site. Although AMR patterns among isolates are often consistent within the same sample and animal, occasional variation might have led to some resistant isolates being missed (29).

The external validity of this study, meaning how generalizable findings are to the target population (58), was considered good. This supports the applicability of the results to Norwegian fattening herds affected by enzootic pneumonia or to herds with similar conditions. However, in herds with low BRD occurrence or in those with different pathogens, breeds, ages, or management practices, the findings may not be directly generalizable.

### 4.6 Conclusion

*P. multocida* and *M. haemolytica* were the most frequently detected BRD-associated bacteria in calves from Norwegian fattening herds with enzootic pneumonia. *P. multocida* appeared most clinically relevant, with detection in BAL strongly associated with clinical disease. Antimicrobial resistance levels among *Pasteurellaceae* spp. were generally low, but still present, emphasizing the continued importance of AM susceptibility testing. Upper airway sampling had limited value for individual diagnosis but may be useful at the herd level when clinically diseased calves are sampled.

## Data Availability

The raw data supporting the conclusions of this article will be made available by the authors, without undue reservation.

## Glossary

Ab-ELISA: Antibody enzyme-linked immunosorbent assay
AM: Antimicrobial
AMR: Antimicrobial resistance
BAL: Bronchoalveolar lavage
BHI: Brain heart infusion
BRD: Bovine respiratory disease
BCoV: Bovine coronavirus
BHV1: Bovine herpesvirus 1
BPI3: Bovine parainfluenza virus 3
BRSV: Bovine respiratory syncytial virus (new name: bovine orthopneumovirus)
BVDV: Bovine viral diarrhea virus
CLSI: Clinical and Laboratory Standards Institute
Ct: Cycle threshold
EMA: European Medicines Agency
EUCAST: European Committee on Antimicrobial Susceptibility Testing
MALDI-TOF MS: Matrix-assisted laser desorption/ionization-time-of-flight mass spectrometry
NMBU: Norwegian University of Life Sciences
NPS: Nasopharyngeal swab
NPV: Negative predictive value
NS: Nasal swab
NVI: Norwegian Veterinary Institute
PPA: Percent positive agreement
PPV: Positive predictive value
qPCR: Quantitative polymerase chain reaction
