# Correction: Cross-sectional study of calves from Norwegian fattening herds with enzootic pneumonia: pathogen occurrence, clinical relevance, antimicrobial resistance, and agreement between respiratory tract sampling sites

**DOI:** 10.3389/fvets.2026.1960336

**Published:** 2026-09-01

**Authors:** Lise Marie Ånestad, Silje Enge Falkeid, Veslemøy Sunniva Oma, Randi Therese Garmo, Amelia R. Woolums, Ane Mohn Bjelland, Maria Stokstad, Thea Blystad Klem

**Affiliations:** 1 Norwegian Veterinary Institute, Ås, Norway; 2 Department of Production Animal Clinical Sciences, Norwegian University of Life Sciences, Ås, Norway; 3 Department of Research and Development, Farm Advisory Services, TINE SA, BTB-NMBU, Ås, Norway; 4 Department of Pathobiology and Population Medicine, Mississippi State University, Mississippi State, MS, United States; 5 Department of Bacteriology, Norwegian Institute of Public Health, Oslo, Norway

**Keywords:** antimicrobial susceptibility, bovine respiratory disease (BRD), bronchoalveolar lavage (BAL), diagnostics, *Mannheimia haemolytica*, nasal swab, nasopharyngeal swab, *Pasteurella multocida*

There was a mistake in Table 4 as published. The 95% confidence intervals (CIs) for the percent positive agreement were incorrectly reported in certain rows. The corrected Table 4 appears below. These corrections do not affect the interpretation or conclusions of the article.

**Table 4:** Agreement and predictive values of NS and NPS culture results relative to BAL for detection of *Pasteurellaceae* species in 86 calves from seven fattening herds.

Population of calves	Health status	Species	Upper airway site	Calves (n) per combination (BAL/upper airway site)				Percent positive agreement (95% CI)^a^	Kappa (95% CI)	Classification	*P* ^b^	PPV %	NPV %
				+/+	+/–	–/+	–/–						
Fattening	Total (*n* = 86)	*P. multocida*	NS	23	24	15	24	54 (44–65)	0.10 (0.00–0.21)	Slight	0.20	61	50
			NPS	29	18	11	28	67 (57–77)	0.33 (0.22–0.44)	Fair	0.26	73	61
		*M. haemolytica*	NS	21	2	37	26	52 (41–63)	0.22 (0.14–0.30)	Fair	0.00	36	93
			NPS	21	2	29	34	58 (46–69)	0.33 (0.24–0.42)	Fair	0.00	42	94
		*H. somni*	NS	6	7	7	66	46 (27–65)	0.37 (0.26–0.47)	Fair	1.00	46	90
			NPS	4	9	12	61	28 (11–44)	0.13 (0.02–0.24)	Slight	0.66	25	87
Fattening and dairy	Total (*n* = 199)	*P. multocida*	NS	47	30	44	78	56 (49–64)	0.24 (0.17–0.31)	Fair	0.13	52	72
			NPS	52	25	36	86	63 (56–70)	0.37 (0.30–0.44)	Fair	0.20	59	77
	Healthy (*n* = 92)	*P. multocida*	NS	17	5	22	48	56 (43–68)	0.36 (0.27–0.46)	Fair	0.00	44	91
			NPS	17	5	19	51	59 (46–71)	0.41 (0.31–0.51)	Moderate	0.01	47	91
	Sick (*n* = 107)	*P. multocida*	NS	30	25	22	30	56 (47–66)	0.12 (0.03–0.22)	Slight	0.77	58	55
			NPS	35	20	17	35	65 (56–74)	0.31 (0.21–0.41)	Fair	0.74	67	64

The table also presents results for detection of *P. multocida* in healthy and diseased fattening and dairy calves combined, including 26 healthy and 60 diseased calves from seven fattening herds, and 66 healthy and 47 diseased calves from nine dairy herds. NS, nasal swab; NPS, nasopharyngeal swab; BAL, bronchoalveolar lavage; PPV, positive predictive value; NPV, negative predictive value.

^a^The 95% confidence interval (CI) was calculated using the Wald (normal approximation) method.

^b^*p*-value is based on McNemar's exact test.

The original version of this article has been updated.

