# Supplementary material for: Cross-sectional study of calves from Norwegian fattening herds with enzootic pneumonia: pathogen occurrence, clinical relevance, antimicrobial resistance, and agreement between respiratory tract sampling sites
Source: Front Vet Sci. 2026 Jun 24;13:1824642. doi: 10.3389/fvets.2026.1824642 (PMC13343233; doi:10.3389/fvets.2026.1824642)
Supplement: Supplementary file 1 [file Table_1.docx]

Supplementary Material

**Table S1.** Key information from nine dairy and seven fattening herds with enzootic pneumonia.

| **Farm data** | | | | | |
| --- | --- | --- | --- | --- | --- |
| **Background** | | | **Calves sampled** | | |
| **Production type** | **Farm ID** | **Region in Southern Norway** | **No. of calves sampled (no. of pens)^1^** | **Age (days)**  Median (range) | **Weight (kg)^2^**  Median (range) |
| Dairy | A | South-east | 20 (4) | 83 (11-293) | 111 (46-291) |
|  | B | South-east | 20 (3) | 76.5 (25-107) | 95 (63-119) |
|  | C | South-west | 15 (5) | 82 (30-116) | 107 (67-160) |
|  | D | North-west | 17 (3) | 50 (33-68) | 69 (50-95) |
|  | E | South-east | 11 (5) | 64 (11-85) | 81 (61-110) |
|  | F | North-east | 12 (2) | 66 (22-86) | 84 (63-92) |
|  | G | North-east | 12 (2) | 60 (19-142) | 80 (50-116) |
|  | H | South-east | 12 (2) | 54 (13-98) | 87 (51-119) |
|  | I | North-west | 12 (1) | 55.5 (30-82) | 77 (65-107) |
|  | **Total (A-I)** |  | **131 (27)** | **64 (11-293)** | **83 (46-291)** |
| Fattening | J | South-east | 16 (1) | 147 (83-217) | 139 (100-190) |
|  | K | South-west | 15 (2) | 136 (124-241) | 142 (107-215) |
|  | L | South-east | 11 (1) | 144 (109-160) | 150 (119-206) |
|  | M | South-east | 4 (1) | 76.5 (58-139) | 84 (79-98) |
|  | N | South-east | 10 (2) | 184 (155-257) | 174 (131-198) |
|  | O | North-west | 12 (3) | 59 (20-96) | 80 (61-100) |
|  | P | South-east | 20 (1) | 135 (107-207) | 139 (119-210) |
|  | **Total (J-P)** |  | **88 (11)** | **138 (20-257)** | **140 (61-215)** |

^1^All sampled pens were located in the same building in each herd and in close proximity to each other, except in herd B where pens were in separate locations of the same barn. ^2^Weight of calves was measured using a weight tape.
