# Supplementary material for: Cross-sectional study of calves from Norwegian fattening herds with enzootic pneumonia: pathogen occurrence, clinical relevance, antimicrobial resistance, and agreement between respiratory tract sampling sites
Source: Front Vet Sci. 2026 Jun 24;13:1824642. doi: 10.3389/fvets.2026.1824642 (PMC13343233; doi:10.3389/fvets.2026.1824642)
Supplement: Supplementary file 2 [file Table_2.docx]

Supplementary Material

**Table S2.** Pre-visit questionnaire form used in farmer interviews.

|  | **Question** |
| --- | --- |
| **Participation** | - Would you like to participate in this project? - Are you a member of the Beef Cattle Performance Recording System (*Storfekjøttkontrollen*) |
| **Production** | - Could you describe the herd and the type of production you have?   - - Is this exclusively a fattening herd?     - How large is the herd?     - How many operational buildings are used for the herd? - Can you describe the housing conditions and feeding routines for the different age groups in the herd, particularly for the calves? - How many farms have you purchased cattle from during the last year?   - - Do you take into account the BRSV/BCoV status^1^ of supplier herd?     - What breeds do you purchase?     - How old are the calves at arrival?     - How many calves do you purchase at once? - What routines do you have for new calf arrivals?   - - Do you quarantine new arrivals?     - Can you describe the housing conditions and feeding routines for calves in quarantine?     - Do you vaccinate new arrivals? If yes, what vaccination regimen do you use?     - How are calves housed and fed post-quarantine? How many calves are kept per pen? |
| **Health** | - Can you describe the occurrence of BRD in the herd in general and over the past two years?   - - Do you have the impression of a persisting BRD problem in the herd? Roughly how many calves are treated yearly? Which age groups are treated? - How has the occurrence of BRD changed? Is the problem increasing or decreasing? - Is BRD a seasonal problem in the herd? - To what extent do you notice mild cough in the calves throughout the year that does not warrant veterinary treatment? - Can you describe the occurrence of other health challenges in the herd, both in general and over the past two years? - Can you describe the antimicrobial use in the herd?   - - Which antimicrobial drugs are used in the herd? - Has anyone tried to solve the BRD issue in the herd? - Does the herd have regular visits from a veterinary practitioner or health advisor? |
| **Other** | - Is there any other relevant information you would like to add? |

^1^In the time period 2016-2022, a nationwide control program led by the national cattle industry encouraged antibody testing for BRSV and BCoV to classify herds as virus positive or negative. This was done to prevent introduction of viruses via live animal sales (1).

**References**

1. Stokstad M, Klem TB, Myrmel M, Oma VS, Toftaker I, Østerås O, et al. Using Biosecurity Measures to Combat Respiratory Disease in Cattle: The Norwegian Control Program for Bovine Respiratory Syncytial Virus and Bovine Coronavirus. Frontiers in Veterinary Science. 2020;7.
