# Supplementary material for: Cross-sectional study of calves from Norwegian fattening herds with enzootic pneumonia: pathogen occurrence, clinical relevance, antimicrobial resistance, and agreement between respiratory tract sampling sites
Source: Front Vet Sci. 2026 Jun 24;13:1824642. doi: 10.3389/fvets.2026.1824642 (PMC13343233; doi:10.3389/fvets.2026.1824642)
Supplement: Supplementary file 3 [file Table_3.docx]

Supplementary Material

**Table S3.** On-site observations during herd visit. Reproduced from Ånestad et al. (1), originally published in *BMC Veterinary Research* under the CC BY 4.0 license.

|  | **Observations/measurements** |
| --- | --- |
| **Anamnesis** | Calf management routines |
|  | Impression of BRD problem (farmer’s and investigator’s observations) |
| **Housing** | Number of individuals per pen |
|  | Age range per pen |
|  | All-in/all-out system (yes or no) |
|  | Hygienic conditions |
|  | Flooring |
| **Feeding routines** | Milk feeding routines |
|  | Type and amount of roughage and concentrate |
| **Bedding material** | Type, amount, and quality |
| **Environmental measurements** | Temperature |
|  | Humidity |
|  | Air quality and the presence of drafts |

1. Ånestad LM, Falkeid SE, Oma VS, Garmo RT, Bjelland AM, Woolums AR, et al. Cross-sectional study of calves from Norwegian dairy herds with enzootic pneumonia: pathogen occurrence, antimicrobial resistance, culture result interpretation, and sampling site agreement. BMC Veterinary Research. 2026.
