# Supplementary material for: Cross-sectional study of calves from Norwegian fattening herds with enzootic pneumonia: pathogen occurrence, clinical relevance, antimicrobial resistance, and agreement between respiratory tract sampling sites
Source: Front Vet Sci. 2026 Jun 24;13:1824642. doi: 10.3389/fvets.2026.1824642 (PMC13343233; doi:10.3389/fvets.2026.1824642)
Supplement: Supplementary file 4 [file Table_4.docx]

Supplementary Material

**Table S4.** Antimicrobial panel with interpretative categories and zone diameter breakpoints for *Pasteurellaceae*.

|  |  | **Inhibition zone diameters (mm)** | | |  |
| --- | --- | --- | --- | --- | --- |
| **Antimicrobial agent** | **Disc content, µg** | **Susceptible** | **Susceptible, increased exposure** | **Resistant** | **Reference** |
| Benzylpenicillin | 10 | ≥ 17 |  | ≤ 16 | EUCAST, Clinical breakpoints (v14.0), 2024 |
| Amoxicillin-clavulanic acid | 30 | ≥ 15 |  | ≤ 14 | EUCAST, Clinical breakpoints (v14.0), 2024 |
| Trimethoprim + Sulfamethoxazole | 1.25/23.75 | ≥ 23 |  | ≤ 22 | EUCAST, Clinical breakpoints (v14.0), 2024 |
| Tetracycline | 30 | ≥ 24 |  | ≤ 23 | EUCAST, Clinical breakpoints (v14.0), 2024 |
| Enrofloxacin | 5 | ≥ 21 | 17-20 | ≤ 16 | CLSI, VET01S ED7, 2024 |
| Florfenicol | 30 | ≥ 19 | 15-18 | ≤ 14 | CLSI, VET01S ED7, 2024 |
| Streptomycin^1^ | 10 | ≥ 15 | 12-14 | ≤ 11 | CLSI, M100-ED34, 2024 |

Abbreviations: EUCAST = European Committee on Antimicrobial Susceptibility Testing; CLSI = Clinical and Laboratory Standards Institute. ^1^For streptomycin, EUCAST and CLSI do not define breakpoints for *Pasteurellaceae* and we therefore applied those of *Enterobacteriaceae* instead, as done in another study (1). Reproduced from Ånestad et al. (2), originally published in *BMC Veterinary Research* under the CC BY 4.0 license.

**References**

1. Maynou G, Bach A, Terré M. Feeding of waste milk to Holstein calves affects antimicrobial resistance of Escherichia coli and Pasteurella multocida isolated from fecal and nasal swabs. Journal of Dairy Science. 2017;100(4):2682–94.

2. Ånestad LM, Falkeid SE, Oma VS, Garmo RT, Bjelland AM, Woolums AR, et al. Cross-sectional study of calves from Norwegian dairy herds with enzootic pneumonia: pathogen occurrence, antimicrobial resistance, culture result interpretation, and sampling site agreement. BMC Veterinary Research. 2026.
