# Supplementary material for: Cross-sectional study of calves from Norwegian fattening herds with enzootic pneumonia: pathogen occurrence, clinical relevance, antimicrobial resistance, and agreement between respiratory tract sampling sites
Source: Front Vet Sci. 2026 Jun 24;13:1824642. doi: 10.3389/fvets.2026.1824642 (PMC13343233; doi:10.3389/fvets.2026.1824642)
Supplement: Supplementary file 5 [file Table_5.docx]

Supplementary Material

**Table S5.** Mean percentage fluid return relative to instilled volume during BAL per culture result. The table includes results from 77 calves from seven fattening herds. The remaining nine fattening calves in this study were excluded from this analysis: four due to missing data and five because they received a second instilment (total 120ml) after an initial failed return.

| **Culture result for *Pasteurellaceae* spp.** | **n** | **BAL fluid return (%)^1^**  **Mean ± SD (range)** |
| --- | --- | --- |
| **Total (all cultures)** | 77 | 31 ± 12 (10-57) |
| **Culture negative** | 26 | 33 ± 14 (10-53) |
| **Culture positive, total** | 51 | 30 ± 11 (10-57) |
| **Culture positive, < 10 colonies** | 24 | 33 ± 12 (10-57) |
| **Culture positive, ≥ 10 colonies** | 27 | 27 ± 11 (10-52) |

Abbreviation: SD = Standard deviation. ^1^Instilled volume was 60ml.
