# Supplementary material for: Cross-sectional study of calves from Norwegian fattening herds with enzootic pneumonia: pathogen occurrence, clinical relevance, antimicrobial resistance, and agreement between respiratory tract sampling sites
Source: Front Vet Sci. 2026 Jun 24;13:1824642. doi: 10.3389/fvets.2026.1824642 (PMC13343233; doi:10.3389/fvets.2026.1824642)
Supplement: Supplementary file 6 [file Table_6.docx]

Supplementary Material

**Table S6.** Distribution per herd of *Pasteurellaceae* species among 26 healthy and 62 diseased calves from seven fattening herds.

| Herd | Culture result detected^1^ | Percentages (n) of each culture result | | | | | | | | | | | | | | | | | | |
| --- | --- | --- | --- | --- | --- | --- | --- | --- | --- | --- | --- | --- | --- | --- | --- | --- | --- | --- | --- | --- |
|  |  | **Calf^2^**  *N = 88; 26 H, 62 D* | | |  | **NS**  *N = 88; 26 H, 62 D* | | |  | **NPS**  *N = 88; 26 H, 62 D* | | |  | **BAL**  *N = 86; 26 H, 60 D* | | |  | **Enriched BAL^3^**  *N = 25; 15 H, 10 D* | | |
|  |  | **Total** | **H** | **D** |  | **Total** | **H** | **D** |  | **Total** | **H** | **D** |  | **Total** | **H** | **D** |  | **Total** | **H** | **D** |
| J | *P. multocida* | 50%  (8/16) | 36%  (4/11) | 80%  (4/5) |  | 19%  (3/16) | 9%  (1/11) | 40%  (2/5) |  | 25%  (4/16) | 18%  (2/11) | 40%  (2/5) |  | 25%  (4/16) | 9%  (1/11) | 60%  (3/5) |  | 30%  (3/10) | 25%  (2/8) | 50%  (1/2) |
|  | *M. haemolytica* | 69%  (11/16) | 55%  (6/11) | 100%  (5/5) |  | 50%  (8/16) | 36%  (4/11) | 80%  (4/5) |  | 50%  (8/16) | 55%  (6/11) | 40%  (2/5) |  | 13%  (2/16) | 9%  (1/11) | 20%  (1/5) |  | 10%  (1/10) | 0%  (0/8) | 50%  (1/2) |
|  | No *Pasteurellaceae* | 19%  (3/16) | 27%  (3/11) | 0%  (0/5) |  | 44%  (7/16) | 64%  (7/11) | 0%  (0/5) |  | 38%  (6/16) | 36%  (4/11) | 40%  (2/5) |  | 69%  (11/16) | 82%  (9/11) | 40%  (2/5) |  | 70%  (7/10) | 75%  (6/8) | 50%  (1/2) |
| K | *P. multocida* | 80%  (12/15) | 40%  (2/5) | 100%  (10/10) |  | 53%  (8/15) | 20%  (1/5) | 70%  (7/10) |  | 60%  (9/15) | 20%  (1/5) | 80%  (8/10) |  | 53%  (8/15) | 0%  (0/5) | 80%  (8/10) |  | 33%  (2/6) | 0%  (0/4) | 100%  (2/2) |
|  | *M. haemolytica* | 67%  (10/15) | 80%  (4/5) | 60%  (6/10) |  | 47%  (7/15) | 40%  (2/5) | 50%  (5/10) |  | 33%  (5/15) | 40%  (2/5) | 30%  (3/10) |  | 7%  (1/15) | 20%  (1/5) | 0%  (0/10) |  | 17%  (1/6) | 25%  (1/4) | 0%  (0/2) |
|  | No *Pasteurellaceae* | 7%  (1/15) | 20%  (1/5) | 0%  (0/10) |  | 13%  (2/15) | 40%  (2/5) | 0%  (0/10) |  | 20%  (3/15) | 40%  (2/5) | 10%  (1/10) |  | 40%  (6/15) | 80%  (4/5) | 20%  (2/10) |  | 50%  (3/6) | 75%  (3/4) | 0%  (0/2) |
| L | *P. multocida* | 91%  (10/11) | 100%  (3/3) | 88%  (7/8) |  | 73%  (8/11) | 100%  (3/3) | 63%  (5/8) |  | 36%  (4/11) | 67%  (2/3) | 25%  (2/8) |  | 27%  (3/11) | 33%  (1/3) | 25%  (2/8) |  | 20%  (1/5) | 100%  (1/1) | 0%  (0/4) |
|  | *M. haemolytica* | 91%  (10/11) | 100%  (3/3) | 88%  (7/8) |  | 82%  (9/11) | 67%  (2/3) | 88%  (7/8) |  | 55%  (6/11) | 67%  (2/3) | 50%  (4/8) |  | 18%  (2/11) | 33%  (1/3) | 13%  (1/8) |  | 0%  (0/5) | 0%  (0/1) | 0%  (0/4) |
|  | *H. somni* | 45%  (5/11) | 33%  (1/3) | 50%  (4/8) |  | 27%  (3/11) | 0%  (0/3) | 38%  (3/8) |  | 27%  (3/11) | 33%  (1/3) | 25%  (2/8) |  | 18%  (2/11) | 33%  (1/3) | 13%  (1/8) |  | 0%  (0/5) | 0%  (0/1) | 0%  (0/4) |
|  | No *Pasteurellaceae* | 0%  (0/11) | 0%  (0/3) | 0%  (0/8) |  | 0%  (0/11) | 0%  (0/3) | 0%  (0/8) |  | 9%  (1/11) | 0%  (0/3) | 13%  (1/8) |  | 55%  (6/11) | 33%  (1/3) | 63%  (5/8) |  | 80%  (4/5) | 0%  (0/1) | 100%  (4/4) |
| M | *P. multocida* | 50%  (2/4) | 100%  (1/1) | 33%  (1/3) |  | 50%  (2/4) | 100%  (1/1) | 33%  (1/3) |  | 50%  (2/4) | 100%  (1/1) | 33%  (1/3) |  | 25%  (1/4) | 0%  (0/1) | 33%  (1/3) |  | 0%  (0/3) | 0%  (0/1) | 0%  (0/2) |
|  | *M. haemolytica* | 75%  (3/4) | 100%  (1/1) | 67%  (2/3) |  | 75%  (3/4) | 100%  (1/1) | 67%  (2/3) |  | 50%  (2/4) | 100%  (1/1) | 33%  (1/3) |  | 0%  (0/4) | 0%  (0/1) | 0%  (0/3) |  | 33%  (1/3) | 100%  (1/1) | 0%  (0/2) |
|  | No *Pasteurellaceae* | 25%  (1/4) | 0%  (0/1) | 33%  (1/3) |  | 25%  (1/4) | 0%  (0/1) | 33%  (1/3) |  | 50%  (2/4) | 0%  (0/1) | 67%  (2/3) |  | 75%  (3/4) | 100%  (1/1) | 67%  (2/3) |  | 67%  (2/3) | 0%  (0/1) | 100%  (2/2) |
| N | *P. multocida* | 100%  (10/10) | 100%  (2/2) | 100%  (8/8) |  | 70%  (7/10) | 100%  (2/2) | 63%  (5/8) |  | 90%  (9/10) | 100%  (2/2) | 88%  (7/8) |  | 100%  (10/10) | 100%  (2/2) | 100%  (8/8) |  | N/A | N/A | N/A |
|  | *M. haemolytica* | 70%  (7/10) | 100%  (2/2) | 63%  (5/8) |  | 70%  (7/10) | 100%  (2/2) | 63%  (5/8) |  | 60%  (6/10) | 100%  (2/2) | 50%  (4/8) |  | 40%  (4/10) | 50%  (1/2) | 38%  (3/8) |  | N/A | N/A | N/A |
|  | *H. somni* | 10%  (1/10) | 0%  (0/2) | 13%  (1/8) |  | 0%  (0/10) | 0%  (0/2) | 0%  (0/8) |  | 10%  (1/10) | 0%  (0/2) | 13%  (1/8) |  | 0%  (0/10) | 0%  (0/2) | 0%  (0/8) |  | N/A | N/A | N/A |
|  | No *Pasteurellaceae* | 0%  (0/10) | 0%  (0/2) | 0%  (0/8) |  | 0%  (0/10) | 0%  (0/2) | 0%  (0/8) |  | 10%  (1/10) | 0%  (0/2) | 13%  (1/8) |  | 0%  (0/10) | 0%  (0/2) | 0%  (0/8) |  | N/A | N/A | N/A |
| O | *P. multocida* | 75%  (9/12) | 100%  (3/3) | 67%  (6/9) |  | 50%  (6/12) | 100%  (3/3) | 33%  (3/9) |  | 42%  (5/12) | 100%  (3/3) | 22%  (2/9) |  | 58%  (7/12) | 67%  (2/3) | 56%  (5/9) |  | 0%  (0/1) | 0%  (0/1) | N/A |
|  | *M. haemolytica* | 83%  (10/12) | 33%  (1/3) | 100%  (9/9) |  | 50%  (6/12) | 0%  (0/3) | 67%  (6/9) |  | 58%  (7/12) | 33%  (1/3) | 67%  (6/9) |  | 42%  (5/12) | 0%  (0/3) | 56%  (5/9) |  | 0%  (0/1) | 0%  (0/1) | N/A |
|  | *H. somni* | 75%  (9/12) | 67%  (2/3) | 78%  (7/9) |  | 42%  (5/12) | 33%  (1/3) | 44%  (4/9) |  | 42%  (5/12) | 67%  (2/3) | 33%  (3/9) |  | 42%  (5/12) | 0%  (0/3) | 56%  (5/9) |  | 0%  (0/1) | 0%  (0/1) | N/A |
|  | No *Pasteurellaceae* | 0%  (0/12) | 0%  (0/3) | 0%  (0/9) |  | 0% (0/12) | 0%  (0/3) | 0%  (0/9) |  | 17% (2/12) | 0%  (0/3) | 22%  (2/9) |  | 17% (2/12) | 33%  (1/3) | 11%  (1/9) |  | 100%  (1/1) | 100%  (1/1) | N/A |
| P | *P. multocida* | 80%  (16/20) | 0%  (0/1) | 84%  (16/19) |  | 20%  (4/20) | 0%  (0/1) | 21%  (4/19) |  | 40%  (8/20) | 0%  (0/1) | 42%  (8/19) |  | 78%  (14/18) | 0%  (0/1) | 82%  (14/17) |  | N/A | N/A | N/A |
|  | *M. haemolytica* | 100%  (20/20) | 100%  (1/1) | 100%  (19/19) |  | 100%  (20/20) | 100%  (1/1) | 100%  (19/19) |  | 90%  (18/20) | 100%  (1/1) | 89%  (17/19) |  | 50%  (9/18) | 100%  (1/1) | 47%  (8/17) |  | N/A | N/A | N/A |
|  | *H. somni* | 60% (12/20) | 0%  (0/1) | 63%  (12/19) |  | 25%  (5/20) | 0%  (0/1) | 26%  (5/19) |  | 40%  (8/20) | 0%  (0/1) | 42%  (8/19) |  | 33%  (6/18) | 0%  (0/1) | 35%  (6/17) |  | N/A | N/A | N/A |
|  | No *Pasteurellaceae* | 0%  (0/20) | 0%  (0/1) | 0%  (0/19) |  | 0%  (0/20) | 0%  (0/1) | 0%  (0/19) |  | 0%  (0/20) | 0%  (0/1) | 0%  (0/19) |  | 6%  (1/18) | 0%  (0/1) | 6%  (1/17) |  | N/A | N/A | N/A |

Abbreviations: NS = nasal swab; NPS = nasopharyngeal swab; BAL = bronchoalveolar lavage; H = healthy; D = diseased. ^1^*Pasteurellaceae* spp. were only listed in a herd in this table if ≥ 1 calf in the herd was positive for a given species. ^2^Calf positive = animals with ≥ 1 sampling site (NS, NPS, or BAL, including enriched samples) positive. ^3^Enriched BAL = BAL fluid enriched with BHI broth, following bacteria negative culture from the corresponding non-enriched BAL fluid, providing additional isolates.
