# Supplementary material for: Cross-sectional study of calves from Norwegian fattening herds with enzootic pneumonia: pathogen occurrence, clinical relevance, antimicrobial resistance, and agreement between respiratory tract sampling sites
Source: Front Vet Sci. 2026 Jun 24;13:1824642. doi: 10.3389/fvets.2026.1824642 (PMC13343233; doi:10.3389/fvets.2026.1824642)
Supplement: Supplementary file 7 [file Table_7.docx]

Supplementary Material

**Table S7.** Univariable analyses of associations between health status (0 = healthy, baseline; 1 = diseased) and predictor variables. Analyses were based on 88 calves (86 with BAL), including 26 healthy and 62 diseased calves from seven fattening herds. Herd was included as random effect.

| Bacterial predictor variables^1^ | Level | β ^2^ | OR^2^ | SE^2^ | 95% CI^2^ | *P*^2^ | N^2^ |
| --- | --- | --- | --- | --- | --- | --- | --- |
| *M. haemolytica* detected, NS | Negative (60) | - | - | - | - | - | - |
|  | Positive (28) | 1.18 | 3.26 | 1.81 | 1.10-9.66 | 0.03 | 88 |
| *M. haemolytica* dominant, NS | Negative (48) | - | - | - | - | - | - |
|  | Positive (40) | 0.81 | 2.24 | 1.26 | 0.74-6.77 | 0.15 | 88 |
| *M. haemolytica* dominant, abundant, NS | Negative (50) | - | - | - | - | - | - |
|  | Positive (38) | 0.65 | 1.92 | 1.09 | 0.63-5.86 | 0.25 | 88 |
| *H. somni* detected, NS | Negative (75) | - | - | - | - | - | - |
|  | Positive (13) | 1.55 | 4.70 | 5.33 | 0.51-43.43 | 0.17 | 88 |
| *M. haemolytica* dominant, abundant, NPS | Negative (72) | - | - | - | - | - | - |
|  | Positive (16) | 1.67 | 5.30 | 5.91 | 0.60-47.18 | 0.14 | 88 |
| *P. multocida* detected, BAL | Negative (39) | - | - | - | - | - | - |
|  | Positive (47) | 1.91 | 6.75 | 3.97 | 2.14-21.37 | < 0.01 | 86 |
| *P. multocida* dominant, BAL | Negative (45) | - | - | - | - | - | - |
|  | Positive (41) | 2.73 | 15.36 | 11.90 | 3.37-70.13 | < 0.01 | 86 |
| *P. multocida* pure culture, BAL | Negative (57) | - | - | - | - | - | - |
|  | Positive (29) | 1.80 | 6.07 | 4.51 | 1.41-26.06 | 0.02 | 86 |
| *P. multocida* dominant, abundant, BAL | Negative (59) | - | - | - | - | - | - |
|  | Positive (27) | 2.18 | 8.85 | 7.25 | 1.78-44.10 | < 0.01 | 86 |
| *H. somni* detected, BAL | Negative (73) | - | - | - | - | - | - |
|  | Positive (13) | 1.52 | 4.58 | 5.19 | 0.50-45.26 | 0.18 | 86 |
| **Other predictor variables^1,3,4^** |  |  |  |  |  |  |  |
| Age*^cont^* | - | - | 1.00 | 0.00 | 0.98-1.01 | 0.74 | 88 |
| Gender | Female (11) | - | - | - | - | - | - |
|  | Male (77) | 1.13 | 3.10 | 2.45 | 0.66-14.63 | 0.15 | 88 |

Abbreviations: NS = nasal swab; NPS = nasopharyngeal swab; BAL = bronchoalveolar lavage. ^1^Categorical variables: all bacterial predictor variables (baseline = negative) and gender (baseline = female) ^2^Model output: β = beta coefficient; OR = odds ratio; SE = standard error; CI = confidence interval; P = p-value; N = sample size.^3^Continuous variable: Age^cont^ ^4^Since all except seven calves were ≥ 60 days old, and all except six calves were Norwegian Red, age (categorical) and breed were not tested.
