# Supplementary material for: Cross-sectional study of calves from Norwegian fattening herds with enzootic pneumonia: pathogen occurrence, clinical relevance, antimicrobial resistance, and agreement between respiratory tract sampling sites
Source: Front Vet Sci. 2026 Jun 24;13:1824642. doi: 10.3389/fvets.2026.1824642 (PMC13343233; doi:10.3389/fvets.2026.1824642)
Supplement: Supplementary file 8 [file Table_8.docx]

Supplementary Material

**Table S8.** *P. multocida* positive cultures (%) among calves by health status, production type, herd, and sampling site.

| **Group** | **Production type** | **Herd (number of calves^1^)** | **Positive cultures,**  **% (n)** | | |
| --- | --- | --- | --- | --- | --- |
|  |  |  | **Nasal swab** | **Nasopharyngeal swab** | **Bronchoalveolar lavage** |
| All | Dairy | A (n=10) | 90% (9) | 80% (8) | 60% (6) |
|  |  | B (n=17) | 24% (4) | 24% (4) | 18% (3) |
|  |  | C (n=13) | 15% (2) | 8% (1) | 15% (2) |
|  |  | D (n=17) | 0% (0) | 0% (0) | 0% (0) |
|  |  | E (n=10) | 90% (9) | 100% (10) | 10% (1) |
|  |  | F (n=12) | 100% (12) | 100% (12) | 92% (11) |
|  |  | G (n=10) | 50% (5) | 50% (5) | 30% (3) |
|  |  | H (n=12) | 17% (2) | 8% (1) | 8% (1) |
|  |  | I (n=12) | 83% (10) | 58% (7) | 25% (3) |
|  | Fattening | J (n=16) | 19% (3) | 25% (4) | 25% (4) |
|  |  | K (n=15) | 53% (8) | 60% (9) | 53% (8) |
|  |  | L (n=11) | 73% (8) | 36% (4) | 27% (3) |
|  |  | M (n=4) | 50% (2) | 50% (2) | 25% (1) |
|  |  | N (n=10) | 70% (7) | 90% (9) | 100% (10) |
|  |  | O (n=12) | 50% (6) | 42% (5) | 58% (7) |
|  |  | P (n=18) | 22% (4) | 39% (7) | 78% (14) |
|  |  | **Total (n=199)** | **46% (91)** | **44% (88)** | **39% (77)** |
| Healthy | Dairy | A (n=7) | 86% (6) | 71% (5) | 57% (4) |
|  |  | B (n=15) | 13% (2) | 20% (3) | 20% (3) |
|  |  | C (n=9) | 11% (1) | 11% (1) | 11% (1) |
|  |  | D (n=5) | 0% (0) | 0% (0) | 0% (0) |
|  |  | E (n=4) | 100% (4) | 100% (4) | 0% (0) |
|  |  | F (n=5) | 100% (5) | 100% (5) | 80% (4) |
|  |  | G (n=6) | 50% (3) | 50% (3) | 33% (2) |
|  |  | H (n=8) | 13% (1) | 13% (1) | 0% (0) |
|  |  | I (n=7) | 86% (6) | 43% (3) | 29% (2) |
|  | Fattening | J (n=11) | 9% (1) | 18% (2) | 9% (1) |
|  |  | K (n=5) | 20% (1) | 20% (1) | 0% (0) |
|  |  | L (n=3) | 100% (3) | 67% (2) | 33% (1) |
|  |  | M (n=1) | 100% (1) | 100% (1) | 0% (0) |
|  |  | N (n=2) | 100% (2) | 100% (2) | 100% (2) |
|  |  | O (n=3) | 100% (3) | 100% (3) | 67% (2) |
|  |  | P (n=1) | 0% (0) | 0% (0) | 0% (0) |
|  |  | **Total (n=92)** | **42% (39)** | **39% (36)** | **24% (22)** |
| Sick | Dairy | A (n=3) | 100% (3) | 100% (3) | 67% (2) |
|  |  | B (n=2) | 100% (2) | 50% (1) | 0% (0) |
|  |  | C (n=4) | 25% (1) | 0% (0) | 25% (1) |
|  |  | D (n=12) | 0% (0) | 0% (0) | 0% (0) |
|  |  | E (n=6) | 83% (5) | 100% (6) | 17% (1) |
|  |  | F (n=7) | 100% (7) | 100% (7) | 100% (7) |
|  |  | G (n=4) | 50% (2) | 50% (2) | 25% (1) |
|  |  | H (n=4) | 25% (1) | 0% (0) | 25% (1) |
|  |  | I (n=5) | 80% (4) | 80% (4) | 20% (1) |
|  | Fattening | J (n=5) | 40% (2) | 40% (2) | 60% (3) |
|  |  | K (n=10) | 70% (7) | 80% (8) | 80% (8) |
|  |  | L (n=8) | 63% (5) | 25% (2) | 25% (2) |
|  |  | M (n=3) | 33% (1) | 33% (1) | 33% (1) |
|  |  | N (n=8) | 63% (5) | 88% (7) | 100% (8) |
|  |  | O (n=9) | 33% (3) | 22% (2) | 56% (5) |
|  |  | P (n=17) | 24% (4) | 41% (7) | 82% (14) |
|  |  | **Total (n=107)** | **49% (52)** | **49% (52)** | **51% (55)** |

^1^Each calf was sampled by nasal swab, nasopharyngeal swab, and bronchoalveolar lavage (BAL).
